# Gradient‐Modified Li‐Rich Manganese‐Based Oxides Cathodes with Breakthrough of Kinetic Limitation for High‐Performance All‐Solid‐State Lithium Metal Batteries

**DOI:** 10.1002/adma.202521690

**Published:** 2026-01-28

**Authors:** Ya Chen, Ling Huang, Awais Ghani, Xiaodong Chen, Xin Gao, Tao Meng, Hongchao Sun, Runjing Xu, Peiyi Ji, Xianghong Deng, Long Bao, Shuang Wang, Nana Wang, Gang Tan, Guoxiu Wang, Zhen Huang

**Affiliations:** ^1^ College of Smart Energy Shanghai Jiao Tong University Shanghai China; ^2^ School of Physical Science and Technology ShanghaiTech University Shanghai China; ^3^ Smart Materials for Architecture Research Lab Innovation Center of Yangtze River Delta Zhejiang University Jiashan Zhejiang China; ^4^ Department of Mechanical Engineering City University of Hong Kong Kowloon Hong Kong China; ^5^ School Department of Materials Science Fudan University Shanghai China; ^6^ Center for Clean Energy Technology School of Mathematical and Physical Science Faculty of Science University of Technology Sydney Sydney New South Wales Australia

**Keywords:** all‐solid‐state lithium batteries, gradient‐modified structure, Li‐rich manganese‐based oxides, mechano‐fusion process

## Abstract

The Li‐rich manganese‐based oxides (LRMO) with high operating voltage and large reversible capacities can demonstrate comprehensive advantages of intrinsic safety and high energy density when employed in all‐solid‐state lithium batteries (ASSLBs). However, severe interfacial incompatibility with solid electrolytes (SEs) arising from unstable lattice oxygen and sluggish Li^+^ ionic transport hinders their practical application. In this contribution, the gradient‐modified structure containing S, Zr co‐doping near‐surface and amorphous Zr(SO_4_)_2_ coating is simultaneously established onto LRMO cathode by a one‐step mechano‐fusion process. The synergistic co‐functionalization stabilizes the oxygen framework, enhances charge transport, and suppresses oxygen dimerization under high potential. Besides, the amorphous Zr(SO_4_)_2_ coating evenly adhering onto LRMO bulk ensures long‐term intimate contact with SEs to guarantee electrochemical activities and restrains interfacial parasitic reactions. Consequently, the optimized A‐LRMO cathode exhibits a high discharge capacity of 292 mAh/g at 0.1 C and 81.8% capacity retention after 2000 cycles at 1 C. Pouch cells delivered an areal capacity of 8.5 mAh/cm^2^ with >99.6% Coulombic efficiency. This gradient‐modification strategy offers an effective pathway to improve interfacial stability and accelerate the practical application of LRMO‐based ASSLBs.

## Graphical abstract

The novel gradient‐modified LRMO has been synthesized via a one‐step mechano‐fusion process, with simultaneous S and Zr co‐doping in the near‐surface region and an amorphous coating. The synergistic co‐functionalization stabilizes the oxygen framework, enhances charge transport, and suppresses oxygen dimerization under high potential, which enable improved cathodic compatibility and accelerates the practical application of LRMO‐based ASSLBs.

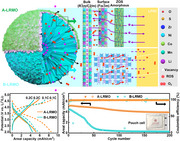

## 1 Introduction

All‐solid‐state lithium batteries (ASSLBs) adopting non‐flammable, leak‐proof, and explosion‐resistant solid electrolytes have garnered widespread attention due to their intrinsic safety, wide operational temperature ranges, and the potential to achieve high energy density [1, 2, 3, 4]. Considering the limitation of practical capacities (<220 mAh/g) and energy densities (<800 Wh kg^−1^) for traditional cathode systems such as LiCoO_2_, Ni‐rich LiNi_(1−x−y)_Co_x_Mn_y_O_2_ and LiFePO_4_, [5, 6, 7] the Li‐rich manganese‐based oxides (LRMO) with high voltage plateau (>4.5 V) [8], superior reversible capacities (>280 mAh/g) and elevated energy densities (>1000 Wh kg^−1^) [9], which are endowed by charge compensation from both transition‐metal ions redox and distinctive lattice oxygen redox, have emerged as promising candidates to fulfill the ambitious requirements of ASSLBs (>500 Wh kg^−1^) and thus further facilitate their application [10], with additional consideration of low‐cost (abundant manganese resources) and non‐toxic characteristics for chief component Mn within LRMO cathode [11, 12].

Notwithstanding these benefits, the LRMO cathode still faces several interconnected challenges that impede its practical application in ASSLBs. First, LRMO exhibits extremely low Li^+^/e^−^ conductivity (σ_Li+_ = ∼10^−11^ S cm^−1^; σ_e−_ = 10^−8^ S cm^−1^) [13], leading to sluggish Li^+^/e^−^ migration kinetics and hindering efficient Li^+^ intercalation/deintercalation during cycling. Second, the space charge layer (SCL) effect, caused by electronegativity differences between solid electrolytes (SEs) and LRMO, increases interfacial resistance and battery polarization. Third, the initial intimate contact between low Young's modulus SEs and LRMO, achieved through lamination, cannot be maintained long‐term due to volume changes during LRMO lithiation/delithiation [14]. This limits the activation of Li_2_MnO_3_ within LRMO cathode, where it is assigned to a unique two‐phase solid solution structure, consisting of LiTMO_2_ and Li_2_MnO_3_ phases, which makes it challenging to fulfill its specific capacities in ASSLBs [15]. Finally, the irreversibility of anions redox feasibly occurs due to metastable oxygen configuration during anions charge compensation process within LRMO, which is more conductive to O─O dimerization and the release of high active oxygen species O^(2−n)−^ (0<n<2) during the electrochemical behaviors. Such these formed species can corrode the SEs for the formation of passivation layer at cathodic interface, thus leading to severe deteriorated interfacial Li^+^ transport kinetics [16]. Additionally, the lattice oxygen vacancies caused by oxygen release could diffuse to the bulk of LRMO and trigger the irreversible phase transition to spinel structure, which is regarded as a primary obstacle to suppress degradation of ASSLBs [17]. Hence, comprehensive consideration of LRMO lattice stability, anionic redox reversibility, cathode interface stability and rapid Li^+^/e^−^ transport kinetic for long term are essential for achieving highly compatible LRMO cathode in ASSLBs.

Up to now, several representative strategies have been executed to response above‐mentioned issues, which mainly concentrate on heteroatoms doping and the architecture of the interfacial coating layer [8]. It is generally acknowledged that the fabrication of interfacial coating layer, such as LiNbO_3_, Li_3_PO_4_, Al_2_O_3_, and LiNb_0.5_Ta_0.5_O_3_, can perform as bridge to mitigate SCL effect through reducing the potential difference between SEs and LRMO cathodes, and simultaneously restrict the SEs parasitic reaction happened at the LRMO/SEs interfaces, thus strengthening interfacial stability [18]. Whereas for heteroatom doping approaches, the incorporation of heteroatoms into LRMO (e.g., B, Si, W, Ru, etc.) can strongly combine with oxygen (O) through covalent bonding [15, 19, 20], which can effectively stabilize the lattice oxygen and minimize local structural change of LRMO. This not only suppresses further electrochemical oxidization of SEs to generate interfacial harmful products, but also prohibits the structural damage through the phase transition from layered to spinel [17]. Besides, such doping methods can regulate the electron configuration to strengthen the Li^+^/e^−^ conductivity of LRMO and meantime construct its high‐efficiency Li^+^/e^−^ transport channels [19], thus contributing to the enhancement of Li^+^/e^−^ transport kinetics. Therefore, combining these two decorative methods will become of great meaningfulness to leverage their respective advantages, and comprehensively address the above‐stated concerns for the application of LRMO materials in ASSLBs, even though the selection of modifier materials compatible with LRMO and the adoption of appropriate preparation methods pose a great challenge.

**FIGURE 1:**
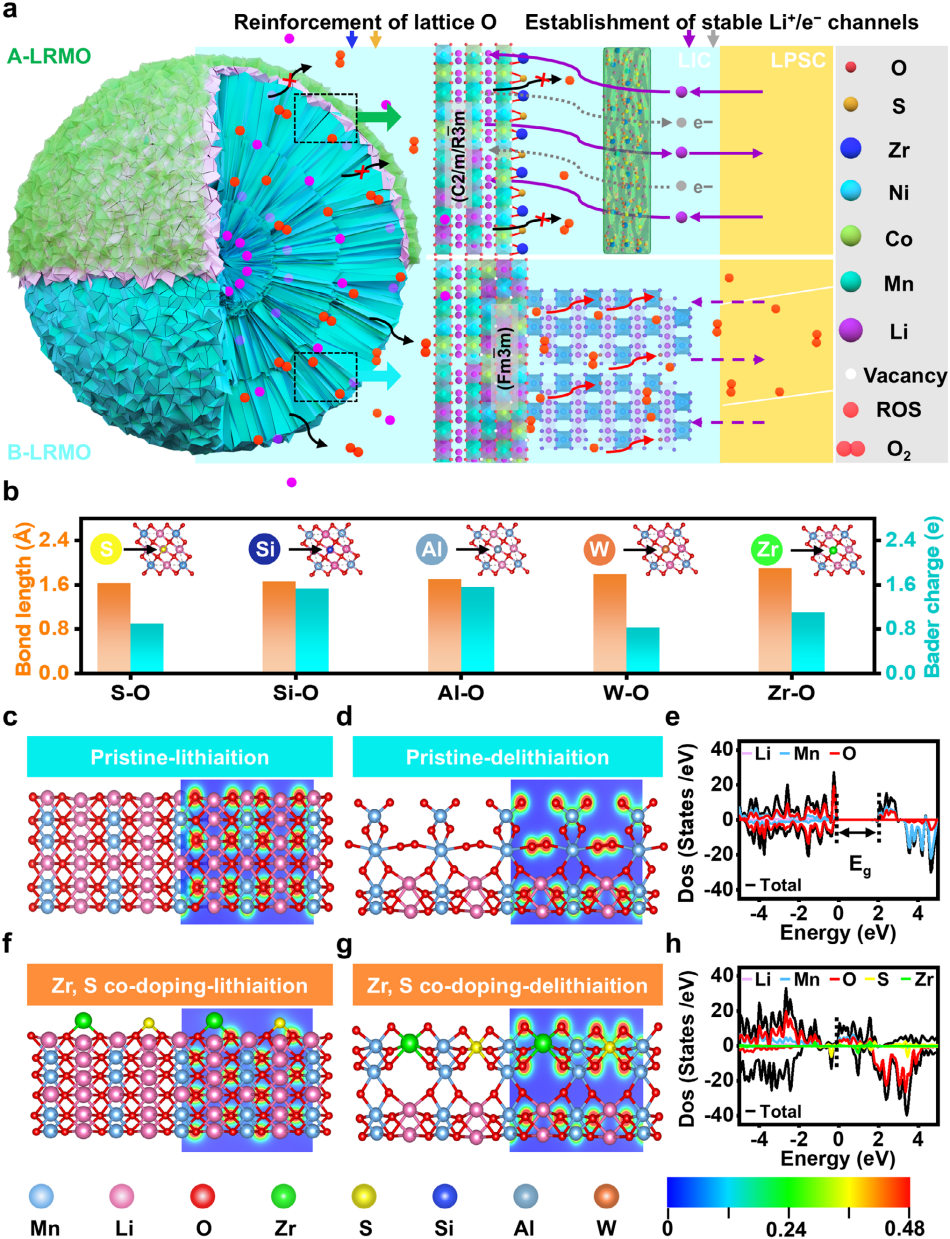
(a) Diagram illustrating the gradient‐modified structure of LRMO on the lattice and cathodic interface stability. DFT calculation analysis. (b) The average bond length and Bader charge of S─O, Si─O, Al─O, W─O, and Zr─O in the LRMO lattice. Contour maps of charge density in (c,d) LRMO, (f,g) Zr‐doping LRMO at lithiation and delithiation states. Calculated density of state (DOS) for (e) LRMO and (h) Zr, S co‐doping LRMO.

In this contribution, to simultaneously balance anion redox activity and reversibility (Bader charge), lattice oxygen stability in LRMO, and rapid Li^+^/e^−^ migration kinetics, zirconium sulfate (Zr(SO_4_)_2_) was preferentially selected as the precursor through theoretical screening of modification elements, and then the gradient‐modified LRMO cathode was efficiently established (labeled as A‐LRMO) through a one‐step mechano‐fusion proceedings, which contains S, Zr co‐doping LRMO bulk and amorphous Zr(SO_4_)_2_ coating. The co‐functionalization of S and Zr modulates local electron configurations and forms strong S/Zr─O covalent bonds with varying lengths, synergistically enhancing Li^+^/e^−^ transport kinetics and lattice oxygen stability (Figure 1a), thereby improving anionic redox activity and reversibility. Additionally, a uniform soft Zr(SO_4_)_2_ coating on LRMO particles ensures long‐term compatibility and rapid Li^+^/e^−^ migration at the cathodic interface, achieving a discharge capacity of 292.04 mAh/g at 0.1C, 81.8% capacity retention after 2000 cycles at 1C, and a lifespan exceeding 5000 cycles. Furthermore, ASSLB pouch cells with an area capacity of 8.5 mAh/cm^2^ maintain stable cycling, retaining 4.18 mAh/cm^2^ after 200 cycles and an average Coulombic efficiency above 99.6%. This discovery offers novel perspectives and strategies for constructing highly compatible interfaces in LRMO cathodes, laying a solid foundation for accelerating the commercialization of ASSLBs.

## 2 Results and Discussion

### 2.1 Element Screening and Computational Validation

Here, we put forward a multifunctional modification strategy combining bulk‐phase doping and surface coating, which represents a critical step toward enabling the stable operation of LRMO with ultrahigh specific capacity in ASSLBs. Thereinto, screening appropriate heteroatoms for doping into LRMO is the first and crucial step during the preparation process, as it mainly holds the key to stabilizing lattice oxygen while enhancing its redox activity and reversibility. Accordingly, DFT calculations were employed to investigate the bond lengths and average Bader charges of S and Zr doping for LRMO. These two parameters are recognized as key characteristics governing the stability of surface oxygen and the reversibility of anionic redox processes. The results of these calculations will be benchmarked against those of the representative Al, Si, and W doping strategies, which have been validated as effective doping modifications for LRMO, owing to their well‐established ability to stabilize lattice oxygen. As shown in Figure 1b, the calculated results are listed as follows: S─O: 1.63 Å, 0.9 e; Si─O: 1.66 Å, 1.53 e; Al─O: 1.70 Å, 1.56 e; W─O: 1.79 Å, 0.83 e; and Zr─O: 1.90 Å, 1.1 e, which demonstrate the clear variation in oxygen bond characteristics induced by different surface functionalization species. Sulfur functionalization with a short S─O bond length contributes to stabilizing the oxygen framework during cycling, thereby suppressing cathodic incompatibility induced by irreversible structural phase change and transition metal diffusion [21]. Simultaneously, the Zr─O bonds formed by Zr functionalization exhibit bond lengths comparable to those of the Mn─O bonds in the host LRMO lattice (1.9 Å). This similarity facilitates the maintenance of Li^+^ transport channels within the lattice, consequently ensuring rapid Li^+^ transport kinetics [22, 23, 24]. Moreover, Bader charge is a crucial parameter for quantifying atomic charge distribution, with its magnitude directly influencing the electron transfer capability of doping atoms and lattice stability. A high Bader charge typically indicates stronger electron transfer capacity, manifesting as enhanced charge compensation capability in LRMO materials. However, excessively high charge values can induce lattice distortion. As shown in Figure 1b, S, Zr co‐doping, compared to these representative heteroatom doping, exhibits moderate average Bader charge values, indicating the formation of a robust covalent bond and enabling the excellent charge compensation capability, which is vital for lattice stability during Li^+^ intercalation/extraction in LRMO materials.

To evaluate the structural evolution of LRMO during the delithiation process, several surface Li^+^ ions were removed to simulate the charged state. As shown in Figure 1c,d, the pristine surface exhibits spontaneous formation of O─O dimers upon delithiation, as evident from the shortened interatomic distance and charge density accumulation between surface O atoms. However, when Zr or S is introduced as a surface functional group individually (Figure S1), no O─O dimers are observed, and the surface oxygen atoms remain well‐separated [25, 26]. In particular, the charge density around oxygen shows polarization toward the functional atoms, suggesting polyanionic interactions that help stabilize the lattice oxygen species [27]. The co‐functionalized surface with both Zr and S (Figure 1f,g) remains stable, with uniformly distributed charge density and complete further suppression of oxygen dimerization. These results confirm that surface functionalization can effectively modulate the local electronic structure and reinforces the O ligand framework, which prevent over‐oxidation of lattice oxygen and strengthen the structural stability during electrochemical cycling. To evaluate the electronic properties of LRMO and its surface‐functionalized variant with Zr, S‐co‐doping, the density of states (DOS) plots in Figure 1e,h are analyzed. Figure 1e shows that pristine LRMO exhibits semiconductive behavior, with a clear bandgap and no states at the Fermi level. Upon surface functionalization with Zr and S (Figure 1h), new states appear near the Fermi level, indicating a significant modification of the electronic structure comparing to that with Zr or S individually (Figure S2). These additional states contribute to increased electronic conductivity, highlighting the impact of Zr and S functionalization on the material's conductive properties. Therefore, through DFT calculations aforementioned, selecting Zr(SO_4_)_2_ as the precursor to achieve coating and co‐doping of LRMO will become of great significance, which will establish a solid theoretical foundation for effective gradient modification of LRMO cathode, as further identified by following detailed experiments and characterizations.

### 2.2 Structural Characterization and Interfacial Li+/E− Transport Dynamics Testing

**FIGURE 2:**
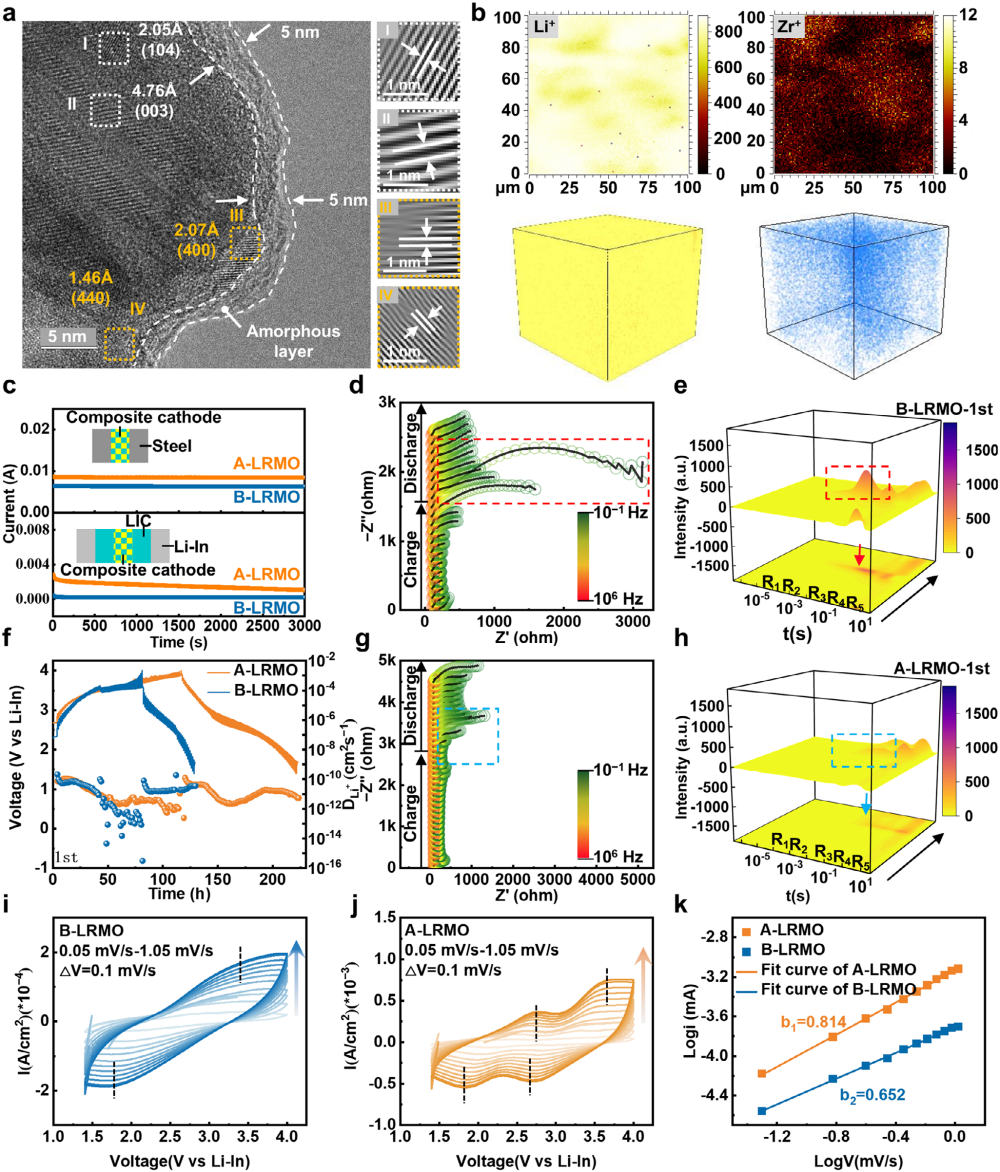
(a) AC‐TEM image of A‐LRMO cathode. (b) Time‐of‐flight secondary ion mass spectrometry (TOF‐SIMS) images of Li^+^ and Zr^+^ species for A‐LRMO cathode. The color bar exhibits the intensity of the ion fragment signals. (c) Current–time curves of the composite cathode under a polarization of 500 mV in a steel/NCM composite/steel ion‐blocking cell and current–time curves of the composite cathode under a polarization of 200 mV in a Li‐In/LIC/composite cathode/LIC/Li‐In electron‐blocking cell for both A‐LRMO and B‐LRMO samples. Galvanostatic electrochemical impedance spectra (GEIS) of (d,e) B‐LRMO‐LIC‐VGCF/LPSC/Li‐In and (g,h) A‐LRMO‐LIC‐VGCF/LPSC/Li‐In ASSLBs during initial cycling accompanied with corresponding contour plots of DRT analyses. (f) Galvanostatic Intermittent Titration Technique (GITT) potential curves of B‐LRMO‐LIC‐VGCF/LPSC/Li‐In and A‐LRMO‐LIC‐VGCF/LPSC/Li‐In ASSLBs during the initial charging and discharging process. CV curves at scan rate from 0.05 to 1.05 mV s^−1^ for (i) B‐LRMO and (j) A‐LRMO cathodes accompanied with (k) b‐values comparison.

Based on the elemental species and relevant precursor Zr(SO_4_)_2_ identified (Figure S3), a well‐defined doping‐coating effect was achieved on LRMO through a simple ball milling process (labeled as A‐LRMO), and the overall phase structure of A‐LRMO does not undergo significant changes, as confirmed by XRD result (Figure S4a) [28]. As shown in Figure 2a, the obvious core–shell structure was revealed by aberration‐corrected transmission electron microscopy (AC‐TEM) images where the crystalline phase is uniformly coated by an amorphous layer with a thickness of around 5–10 nm. In the crystalline regions (Region I and Region II), distinct lattice fringes with interplanar spacings of 2.05 and 4.76 Å are observed, which correspond to the (104) and (003) planes of the layered structure (space group R‐3m) [29]. In contrast, no lattice fringes are visible in the coating layer, which originates from the amorphization process of Zr(SO_4_)_2_ during high‐energy synthesis process, as evidenced by its XRD results showing significantly reduced crystalline peak intensities (Figure S4b). Additionally, the rock‐salt phase structure (space group Fm‐3m) could also be detected with interplanar spacings of 2.07 and 1.46 Å matching the (400) and (440) planes, which are sporadically interposed between layered phase and the amorphous phase [30]. Similarly, for the bare LRMO (B‐LRMO) after ball milling, lattice fringes corresponding to the layered phase structure ((101), (003), and (104) planes) and the rock‐salt phase structure ((222) and (440) planes) are observed in Figure S5, with interplanar spacings of 2.46, 4.76, 2.05, 2.39, and 1.46 Å, respectively, which indicates that high‐energy ball milling process induces the formation of interwoven structure of layered and rock‐salt phases at surface of LRMO cathode [31]. Compared to A‐LRMO, B‐LRMO exhibits more severe phase transformation and a less uniform distribution of rock‐salt phase structure, highlighting the positive role of Zr(SO_4_)_2_ addition for establishment of uniform coating. The atomic‐resolution high‐angle annular dark‐field scanning transmission electron microscopy (HAADF‐STEM) and electron energy loss spectroscopy (EELS) analysis confirm Zr^4^^+^ doping at Li sites in the A‐LRMO cathode. As shown in Figure S6, the material maintains a layered structure with a lattice fringe spacing of 4.78 Å for the (003) plane, slightly larger than the bulk phase spacing (4.76 Å). This expansion results from lattice distortion due to Zr^4+^ doping; although Zr^4+^ and Li^+^ have similar ionic radii (∼0.72 vs. ∼0.76 Å), the higher valence of Zr^4+^ increases its effective coordination radius, and charge compensation generates Li^+^ vacancies that further relax the lattice. Furthermore, distinct bright atomic columns correspond to doped Zr atoms with high atomic contrast comparing to Ni, Co, and Mn, confirming successful incorporation into the near‐surface structure of A‐LRMO (Figure S6a). Additionally, Figures 5Xb shows an energy loss peak at ∼180 eV corresponding to the Zr M_5_ edge, which confirms Zr doping. In Figure 5Xd, despite limited resolution at high magnification, the Zr signal in the EELS elemental mapping is distinctly separated from the Mn signal, also indicating Zr incorporation into lithium layer sites to some extent.

The scanning electron microscope (SEM) observations combined with energy dispersive spectroscopy (EDS) analyses for A‐LRMO cathode confirm the homogeneous coating structure, as evidenced by uniform distributions of O, Mn, Co, Ni, S, and Zr elements (Figure S7). And the heterojunction structure of A‐LRMO cathode was also verified by EDS analyses under linear scanning mode, of which the scanning route was settled across the interface (Figure S8a). As the detection point moves from particle center toward edge, the signal intensities of Zr, S, and Mn elements decrease to varying degrees (Figure S8b). The signal of Mn, representing the LRMO cathode, disappears at approximately 27 nm from the origin, while the signals of Zr and S elements, representing the Zr(SO_4_)_2_ coating layer, persist until about 37 nm. This demonstrates that the Zr(SO_4_)_2_ component extends further at particle edge compared to LRMO cathode, providing strong evidence for the successful construction of the LRMO/Zr(SO_4_)_2_ core–shell structure. Besides, the decrease of Zr and S signal intensities could be observed at the outermost edge of the particle profile, which may be ascribed to the damage of amorphous coating layer under high‐energy electron beam, resulting in the signal attenuation during linear scanning and EDS signal acquisition. In addition, the TOF‐SIMS measurements were also performed to obtain detailed component information of Zr(SO_4_)_2_ amorphous coating. As seen in Figure 2b, the clear distribution profiles for Li^+^ and Zr^+^ fragments collected from A‐LRMO could be detected, manifesting the successful coating of Zr(SO_4_)_2_ component. And 3D reconstruction accompanied also demonstrate the generation of abundant Li^+^ and Zr^+^ fragments with even distributions throughout A‐LRMO powder. To be mentioned, the highly overlapping distribution regions of Li^+^ and Zr^+^ indicate that inevitable interdiffusion occurs between the Zr(SO_4_)_2_ coating layer and LRMO components under high‐energy milling. Consequently, the resulting doping effects on the LRMO crystal lattice necessitate further focused investigation. Hence, the X‐ray photoelectron spectroscopy (XPS) measurements of A‐LRMO, B‐LRMO were performed, and peaks deconvolution of Zr 3d, S 2p, O 1s, and Mn 3s spectra with specific peak positions labeled are shown in Figures S9–S18 and Table S1, which reflects the various oxidation state of elements. For Zr 3d spectra, the doublets located at 184.50/182.00 eV and 183.90/181.50 eV after etching for 0 nm depth and 10 nm depth respectively manifest the successful introduction of Zr^4+^ onto A‐LRMO (Figure S9). Besides, the evident offset toward lower binding energy comparing to that of pure Zr(SO_4_)_2_, which located at 186.33 and 183.95 eV respectively (Figure S10) [32], indicates significant changes of surrounding environment of Zr^4+^. Considering the similar atomic radius between Zr^4+^ and Li^+^ (∼0.72 and ∼0.76 Å, respectively) [33, 34], which differs substantially from that of other constituent elements (Mn^4+^: ∼0.53 Å, Ni^2+^: ∼0.69Å, Co^3+^: ∼0.545 Å), Zr^4+^ are highly likely to incorporate into Li^+^ sites within LRMO lattice, as rationalized by charge compensation theory. Accordingly, the peak for A‐LRMO cathode in Li 1s spectra exerts a characteristic shift toward higher binding energy compared to B‐LRMO counterpart (Figure S11). This phenomenon conclusively verifies that Li^+^ ions from the LRMO lattice could also substitute into Zr^4+^ sites within the amorphous coating layer, subsequently coordinating with SO_4_^2−^ unit. For S 2p spectra, the peaks at 169.43 and 168.23 eV could be ascribed to SO_4_^2−^ unit within coating layer of A‐LRMO cathode after etching for 0 and 10 nm depths.(Figures S12 and S13) [35], showing a slight toward lower binding energy relative to pure Zr(SO_4_)_2_(169.57 and 168.27 eV, respectively) [32], accompanied by increased SO_3_^2−^ peaks located at 167.73 and 166.43 eV. As for O 1s spectra, the signals at 531.97 eV appear for both pure Zr(SO_4_)_2_ and A‐LRMO samples, manifesting the successful coating of Zr(SO_4_)_2_ onto LRMO cathode (Figures S14 and S15). And the peaks densities of physically absorbed O (532.85 eV, labeled as O’) and O vacancy (531.15 eV, labeled as O_v_) for A‐LRMO cathode significantly decrease with the growth of etching depths, which are also less than that within the precursors including Zr(SO_4_)_2_ and B‐LRMO samples [25, 36], manifesting the enhancement of lattice structure and evident purification during mechano‐fusion process. The peak located at 529.77 eV represents lattice O (labeled as TM‐O) within A‐LRMO sample, which keep still with within the range from 0 to 30 nm depths and shows a shift toward higher binding energy compared to that of B‐LRMO sample (529.25 eV). Based on the combined consideration of the S reduction tendency and the elevated valence state of lattice O, the doping of S atoms into LRMO lattice during the high‐energy ball milling process is preliminarily confirmed. During this process, the coordination bonding formed between S atoms and lattice O induces a shift of electron density toward the S atoms, leading to changes in the valence states of both elements. As for Mn 3s spectra, the signals for A‐LRMO cathode mainly split into 2 peaks at 88.81 and 84.10 eV, whereas the peaks for B‐LRMO cathode locate at 88.71 and 83.85 eV (Figure S16). The splitting energies for both A‐LRMO (4.71 eV) and B‐LRMO cathodes (4.86 eV) indicate the mixed‐valence state of Mn^III^ and Mn^IV^, which are coincident with reported references [37]. Especially, the average valence of Mn improves for A‐LRMO cathode compared to that of B‐LRMO cathode, as evidenced by reduced splitting energy, which also originates from the Zr/S doping and electron density variation aforementioned. Besides, the signals for Mn 2p remains unchanged during the etching process within the range of 20 nm (Figure S17). In summary, during the ball‐milling process, Zr and S elements from Zr(SO_4_)_2_ are effectively doped into the near‐surface Li^+^ sites of LRMO cathode, while Li^+^ ions migrate into the Zr(SO_4_)_2_ structure through reciprocal diffusion, which are confirmed by characteristic peak shifts of relevant elements in XPS results. The well‐defined core–shell structure established via mechano‐fusion process features rapid ionic transfer owing to the elevated Li^+^ ions concentration within the Zr(SO_4_)_2_ coating layer, and robust lattice stability from Zr/S doping in the near‐surface region.

To quantitatively investigate the enhancement of electrochemical activity for novel A‐LRMO cathode prepared by mechano‐fusion method, the ionic and electronic conductivities of the composite cathode were tested from a practical application perspective. Specifically, stainless steel discs were pressed as Li^+^ ions blocking electrodes on both sides of composite cathode interlayer, and the electronic conductivity was measured via direct current (DC) polarization testing of assembled sandwich‐structured cell [38, 39]. As shown in Figure 2c and Table S2, the electronic conductivity of composite cathode with B‐LRMO sample is 6.59 × 10^−4^ S/cm, while that of A‐LRMO sample significantly improves to 9.48 × 10^−4^ S/cm. This indicates that Zr(SO_4_)_2_ effectively enhances internal electronic transport within composite cathode rather than acting as an inert material with counterproductive effects, which is crucial for rapid reaction kinetics during electrochemical insertion and extraction of Li^+^ ions within LRMO cathode. The improved electronic conductivity of A‐LRMO powder (1.50 × 10^−4^ S/cm) is also higher than pure Zr(SO_4_)_2_ (7.96 × 10^−5^ S/cm) and B‐LRMO (below the detection limit) (Figure S19), further verifying the synergistic enhancement by gradient modification. For Li^+^ ionic conductivity, the Li‐In/LIC/composite cathode/LIC/Li‐In cell was assembled, with LIC and Li‐In foils as electron‐blocking layer and non‐blocking electrodes, respectively. The Li^+^ ionic conductivity was measured using the same DC polarization method (Figure 2c; Table S3). The A‐LRMO composite reaches 2.91 × 10^−4^ S/cm, whereas the B‐LRMO composite is only 1.98 × 10^−4^ S/cm, and such discrepancy may be attributed to micro‐voids at rigid B‐LRMO/LIC interface. In contrast, the modified A‐LRMO sample features a flexible coating with high ionic conductivity coating that effectively facilitates Li^+^ ionic transfer across the surface. Besides, the value of 2.03 × 10^−3^ S/cm obtained for Li‐In/LIC/Li‐In cell is consistent with the result of EIS test and reported literatures (Figure S20) [40, 41], which confirms the verification of such method.

The significant variations of electrochemical behavior for ASSLBs primarily originate from interfacial kinetic evolution for Li^+^ ions, which can be revealed through in situ galvanostatic electrochemical impedance spectra (GEIS) combined with distribution of relaxation times (DRT) analysis. Assembled A‐LRMO‐LIC‐VGCF/LPSC/Li‐In and B‐LRMO‐LIC‐VGCF/LPSC/Li‐In ASSLBs underwent constant‐current charge/discharge cycling within a voltage range of 2.02–4.62 V (vs. Li^+^/Li) at 0.1 C with impedance spectra collected every 30 min. As shown in Figure 2d, for ASSLB with B‐LRMO cathode during the initial charge to 4.62 V (vs. Li^+^/Li), the interfacial impedance in the mid‐to‐low frequency region increases sharply from 120 to 2850 Ω. In stark contrast, the ASSLB with A‐LRMO cathode exhibits significantly lower interfacial impedance growth under identical conditions, rising only from 130 to 1200 Ω (Figure 2g). To precisely analyze kinetic evolution for Li^+^ ions at cathodic interfaces within ASSLBs given the presence of multiple mass transport interfaces, distribution of relaxation times (DRT) analysis for GEIS was employed due to its unparalleled advantage in differentiating distinct interfacial contributions. The peaks in the range of 10^−5^–10^−4^ s correspond to grain boundary impedance of the SEs (R_1_), and the signals between 10^−4^ and 10^−3^ s originates from solid electrolyte interphase (SEI) formation at the anode side (R_2_). The peaks spanning 10^−3^–10^−1^ s represent the cathodic interface resistance(R_3_), and peaks at 10^−1^–10^0^ s signify charge transfer (CT) at the Li‐In/LPSC and LRMO/LIC interfaces (R_4_). Besides, the signals within 10^0^–10^1^ s are attributed to solid‐state diffusion within the LRMO cathodes (R_5_). As shown in Figure 2e,h, during the charge and discharge process, the R_1_ and R_2_ signals exhibit no significant fluctuations for ASSLBs with both A‐LRMO and B‐LRMO cathodes, which indicates the stability of SEs and Li‐In anodes adopted during cycling, thus not interfering with the analysis of interfacial impedance variations. In the DRT spectra of B‐LRMO‐LIC‐VGCF/LPSC/Li‐In ASSLB during its first charge cycle (Figure 2e), R_3_ and R_4_ increase drastically upon charging into the high‐voltage region, whereas only minimal growth could be observed even at the charge cutoff voltage of 4.62 V (vs. Li/Li^+^) for the cell with A‐LRMO, which demonstrate the suppressed interfacial degradation within A‐LRMO composite cathode and rapid diffusion kinetics for Li^+^ ions maintained even at high voltages. During subsequent discharge process, the degradations induced at high voltages for B‐LRMO composite cathode display irreversibility, as evidenced by large interfacial resistance retained. Conversely, the interfacial impedance largely recovers to its initial state after completion of discharge process for A‐LRMO composite cathode, confirming the markedly enhanced cathodic stability through Zr(SO_4_)_2_ modification, and such enhanced cathodic compatibility could also be maintained at subsequent cycles (Figures S21–S23). In addition, the peaks densities of R_5_ for cell with A‐LRMO cathode remain consistently lower than that of B‐LRMO cathode throughout cycling, suggesting that the doping effect at near‐surface structure of LRMO lattice ensures efficient solid‐state diffusion within bulk cathode. The Galvanostatic Intermittent Titration Technique (GITT) tests were performed to investigate Li^+^ ionic diffusion coefficients (D_Li_^+^) at cathodic interfaces. As shown in Figure 2f, the initial charge–discharge curves for GITT tests and corresponding D_Li_^+^ values are obtained on the basis of Fick's second law [42, 43]. The ASSLB with A‐LRMO cathode exert higher D_Li_^+^ values during charge and discharge processes owing to flexible amorphous coating established with high ionic conductivity as well as superior stability. The less fluctuations and variations of D_Li_^+^ within delithiation and lithiation state are observed for A‐LRMO cathode, and such trend could be maintained in the subsequent cycles compared to B‐LRMO cathode (Figure S24). By contrast, the serious interfacial degradations induced by irreversible mechanical disruptions, chemical/electrochemical parasitic reactions as well as O radicals corrosions, lead to attenuated D_Li_^+^ value during cycling for B‐LRMO sample.

To investigate Li^+^ ions behavior and charge storage mechanisms within composite cathodes, cyclic voltammetry (CV) tests at varying scan rates were conducted on ASSLBs assembled with A‐LRMO and B‐LRMO cathodes. Usually, the total charge storage process comprises two parts: diffusion‐controlled bulk reactions and surface‐related capacitive processes [44].

(1) Logi=loga+blogv

As quantified by Equation (1), the slope value b, which is derived from linear fitting of peak currents vs. scan rates, determines kinetic dominance. As shown in Figure 2i–k, the b‐values of A‐LRMO and B‐LRMO samples are 0.814 and 0.652, respectively, demonstrating the electrochemical reactions of both samples are controlled by combined diffusion and capacitive processes, and the significantly higher b‐value for A‐LRMO sample indicates capacitive dominance attributed to its well‐defined coating [14].

(2) i=k1v+k2v1/2

(3) i/v1/2=k1v1/2+k2

Further quantification via Equations (2) and (3) reveals consistently higher pseudocapacitive contributions for A‐LRMO compared to B‐LRMO sample at all scan rates, and CV curves with capacitive current distinguished from current at 0.95 mV s^−1^ as an example are shown in Figure S25. The A‐LRMO sample with faster pseudocapacitive process demonstrates enhanced interfacial stability and accelerated charge storage kinetics, consequently yielding superior rate capability manifested as higher specific capacity under high‐current conditions.

Based on the comprehensive analysis of morphology, lattice structure, electrochemical activity, and interfacial Li^+^ ions migration behavior, the A‐LRMO cathode with gradient‐modified structure is preliminarily confirmed to exhibit intimate contact and enhanced stability at cathodic interface in ASSLBs as well as rapid Li^+^ ions transfer therein.

**FIGURE 3:**
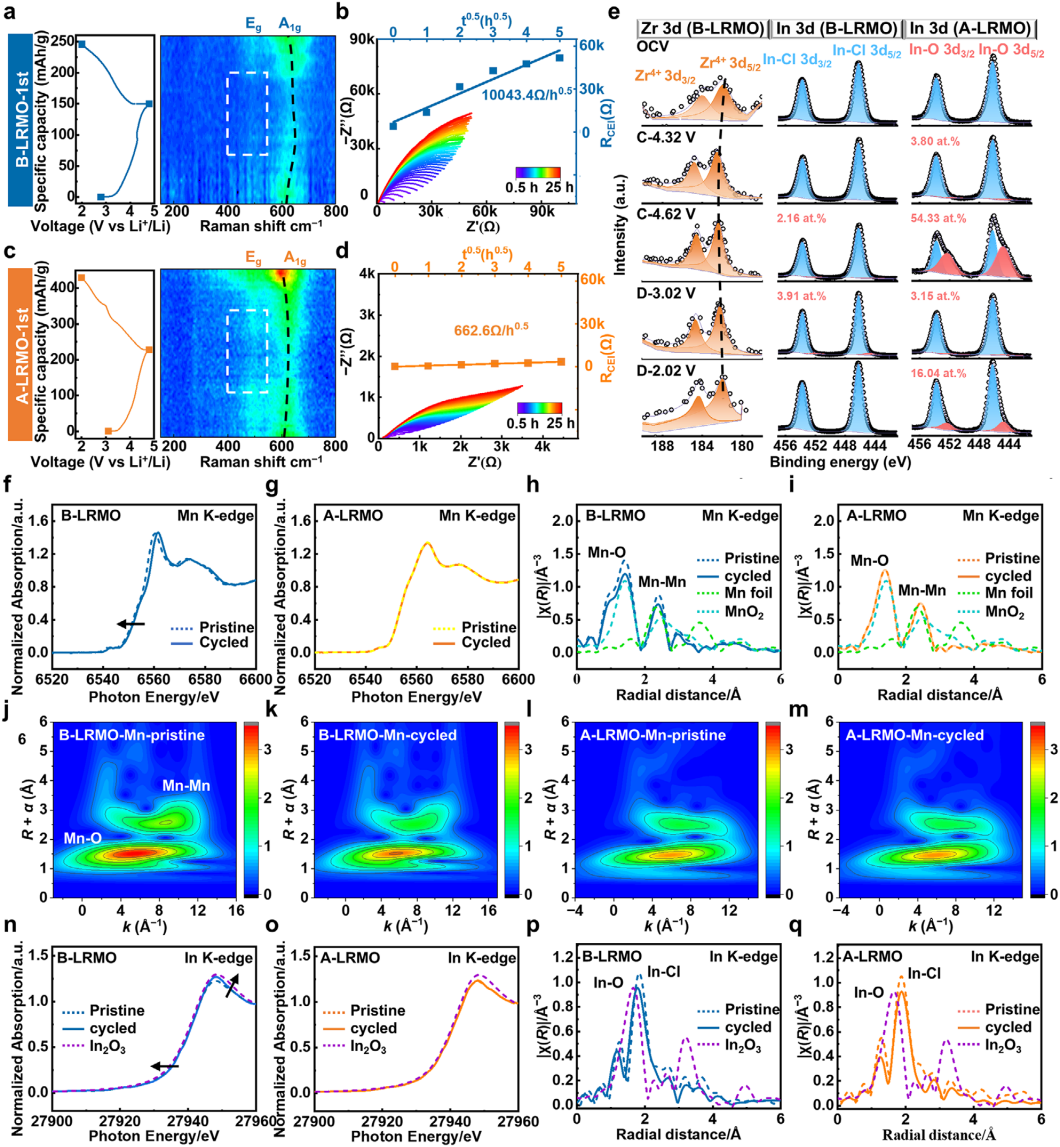
The charge–discharge curves and contour plots of *operando* Raman characterizations for ASSLBs with (a) B‐LRMO and (c) A‐LRMO cathodes. Nyquist plots of (b) B‐LRMO‐LIC‐VGCF/LPSC/Li‐In and (d) A‐LRMO‐LIC‐VGCF/LPSC/Li‐In ASSLBs after charging up to and maintaining at 4.62 V vs. Li+/Li under 0.1C at RT, accompanied with interface resistance of R_CEI_ change as a function of the square root of time (t^0.5^). (e) XPS spectra and curve fitting for Zr 3d and In 3d of A‐LRMO and B‐LRMO cathodes at various states of charge during charge/discharge processes. Normalized XANES spectra and Fourier‐transformed EXAFS spectra at Mn K‐edge of (f,h) B‐LRMO and (g,i) A‐LRMO cathodes before and after cycling with Mn foil and MnO_2_ as references, accompanied with (j–m) corresponding wavelet‐transform images. Normalized XANES spectra and Fourier‐transformed EXAFS spectra at In K‐edge of (n,p) B‐LRMO and (o,q) A‐LRMO cathodes before and after cycling with In_2_O_3_ as reference.

The interface evolution and stability enhancement mechanism of A‐LRMO cathode requires further investigation. Therefore, we conducted *operando* Raman analysis on LRMO cathodes during cycling. As shown in Figure 3a,c, in the initial state of cycling, the signals at around 500 and 600 cm^−1^ could be detected for both A‐LRMO and B‐LRMO samples, which originate from O─Mn─O bend in plane (Eg mode) and Mn─O stretching out of plane (A1g mode), manifesting the characteristic hexagonal O2‐type layered structure maintained [45]. During the initial charge process, the intensities of both characteristic peaks increase, which could be ascribed to the shortening of Mn─O bond with a more intense oxygen vibration during continuous oxidation of Mn [17, 46], And subsequent decrease of Eg and A1g peak intensities at the higher potential could be related to oxidation of lattice oxygen. As seen in Figure 3a,c, the higher intensities of Eg and A1g peaks for A‐LRMO cathode could be observed comparing to that of B‐LRMO cathode during charge process, manifesting superior stability for the lattice structure of A‐LRMO sample, which effectively suppresses the formation of metastable O─O dimers and irreversible phase transition from layered to rock‐salt [46]. Especially, the evident area with low signal intensity appears for B‐LRMO sample, indicating serious lattice collapse and corrosion effect induced by singlet oxygen at high voltage. Besides, the Raman characteristic peaks of A‐LRMO demonstrate superior intensity restoration and reduced positional fluctuation during cycling (Figure S26), indicating its distinct advantages over B‐LRMO in terms of lattice stability and electrochemical lithium (de)intercalation kinetics.

To further investigate the interfacial stability and Li^+^ ion transport kinetics at cathodic side under high voltage, ASSLBs employing A‐LRMO and B‐LRMO cathode materials were charged to 4.62 V vs. Li^+^/Li and held at this potential, and then EIS measurements were conducted every 30 min during this period, precisely capturing the dynamic evolution of chemical degradation at cathodic interface [47, 48]. As shown in Figure 3b,d, the high‐frequency intercept of the impedance spectra with the real axis represents the bulk resistance within SEs (R_SE_), while the distinct semicircle in the mid‐frequency region corresponds to the cathodic interfacial impedance (R_CEI_) [49, 50, 51, 52]. Figure S27 reveals that the R_SE_ for the cell with A‐LRMO remains nearly unchanged over 25 h under high voltage, whereas the cell with B‐LRMO exhibits significant R_SE_ growth (from 108.42 to 126.47 Ω) owing to aggressive corrosion by oxygen radicals formed through irreversible oxidation of lattice oxygen within B‐LRMO cathode. As for cathodic interface, the cell with A‐LRMO exhibits a much slower interfacial impedance increase in R_CEI_ compared to B‐LRMO, attributable to enhanced lattice stability and suppressed oxygen radical evolution achieved through surface functionalization.

(4) $$R_{\mathrm{CEI}}=\frac{1}{S\bar{\sigma_{CEI}}}\sqrt{\frac{V_{m}}{xF^{2}}\cdot \frac{\bar{\sigma_{Li^{+}}}\cdot \bar{\sigma_{e^{-}}}}{\bar{\sigma_{Li^{+}}}+\bar{\sigma_{e^{-}}}}\Delta \mu_{\mathrm{Li}}}\cdot \sqrt{t}\frac{1}{S\bar{\sigma_{CEI}}}\cdot k\sqrt{t}=k^{\prime }\sqrt{t}$$

To quantitatively analyze the interfacial degradation mechanism, the diffusion‐controlled Wagner model was applied to describe the growth of R_CEI_ as Equation (4) with specific parameters labeled in Table S4, where Li^+^ were assumed to be the predominant charge carriers (σ_Li_^+^ >> σ_e_^−^) [53]. The R_CEI_ values plotted against t^0.5^ show that A‐LRMO follows the Wagner model well, whereas B‐LRMO exhibits significant data scattering in the fitting results. Under the controlled experimental conditions, all relevant thermodynamic parameters (including temperature and electrode composition) and lithium chemical potential relating to state of charge are fixed. Hence, the growth of the cathodic degradation layer is driven by the lithium chemical potential difference between SEs and LRMO. And the rate constant k’ for A‐LRMO maintains at a lower level (662.6 Ω/h^0.5^) compared to that for B‐LRMO (10043.4 Ω/h^0.5^), which effectively mitigating chemical degradation, indicating that the highly compatible cathodic interface through surface functionalization has been established for A‐LRMO (Figure S28). Besides, the cathodic stability for both A‐LRMO and B‐LRMO were also tested under open circuit potential after charging up to 4.62 V vs. Li^+^/Li under 0.1C at RT.

Over 50 h, A‐LRMO showed negligible impedance growth, whereas B‐LRMO continued to degrade (Figures S29 and S30). Under open‐circuit voltage conditions, A‐LRMO exhibits negligible interfacial impedance evolution, indicating the formation of a passivation layer that effectively suppresses further parasitic reactions at cathodic interface. In contrast, the previously observed R_CEI_ increase during constant‐voltage operation at 4.62 V vs. Li^+^/Li is attributed to interfacial corrosion induced by lattice oxygen evolution.

### 2.3 Interfacial Stability Testing and Theoretical Validation

To further investigate the interfacial stability of LRMO cathodes at different lithium (de)intercalation states, XPS measurements were performed on the composite cathodes at various states of charge during charge/discharge processes, in which the O 1s, Zr 3d, and In 3d spectra were acquired and deconvoluted for analyzing the components revolution process for both active materials (LRMO) and catholyte (LIC) within composite cathodes (Figure 3e; Figure S31 and Table S1). As shown in Figure S31, the obvious signal of lattice O within B‐LRMO cathode appears, whereas only minor content of lattice O could be detected for A‐LRMO cathode owing to the well‐established overlayer of S─O units. The peak at 530.3 eV appears (5.44 at. %) for B‐LRMO cathode at charge state of 4.62 V vs. Li^+^/Li, indicating substantial irreversible oxidation of lattice O and concomitant vigorous formation of oxygen radicals. By contrast, the components of A‐LRMO cathode including lattice O, O vacancy, S─O bond and physically adsorbed O remain almost unchanged during charge/discharge process, which ensure the chemical and mechanical stability for gradient‐modified structure. Especially in Zr 3d spectra (Figure 3e), the reversible shift of Zr^4+^ doublet, which moves toward higher binding energy during charge and returns upon discharge (Table S5), indicates concerted oxidation between Zr^4+^ and lattice O during Li^+^ (de)intercalation of A‐LRMO cathode, thereby facilitating charge compensation and suppressing lattice oxygen release during cycling. As for In 3d spectra, the doublet at 453.8 and 446.2 eV represents the characteristic signals of In─Cl bond in LIC [54], which is observed for both A‐LRMO and B‐LRMO composite cathodes. During cycling, the prominent occurrence of In─O bond (452.2 and 444.8 eV) at high operating voltage of 4.62 V vs. Li^+^/Li (54.33 at.%) manifests the dramatic formation of oxygen radicals and the corrosion of LIC for B‐LRMO composite cathodes [40], which still remains at the end of discharge process (16.04 at.%). Oppositely, the purity of LIC during cycling maintains at a high level for A‐LRMO composite cathodes with no visible formation of In─O bond, confirming effective suppression of oxygen radicals generation throughout cycling.

X‐ray absorption near edge structure (XANES) and Fourier‐transformed extended X‐ray absorption fine structure (EXAFS) analyses were performed to further investigate the chemical environment of Mn and In atoms, which represent the stability of LRMO and LIC respectively within composite cathodes. For Mn K‐edge XANES spectra, the evident shape change and shift to higher energy occurs for B‐LRMO cathode (Figure 3f), which indicates the irreversible oxidation of Mn and structure transformation relating to immigrated Mn ions and oxygen release. In contrast, A‐LRMO cathode exhibits negligible change after cycling (Figure 3g), implying the well‐maintained local environment around Mn. And such stable valences for Mn element in A‐LRMO cathode demonstrate the high reversibility of lithiation/delithiation processes and steady cation‐anion redox couples, which manifests the establishment of cathodic interface with high compatibility [10]. Furthermore, in Fourier‐transformed EXAFS spectra of Mn K‐edge (Figure 3h,i), two obvious peaks at around 1.5 and 2.4 Å correspond to Mn─O shell and Mn─Mn shell along ab‐plane respectively for both A‐LRMO and B‐LRMO sample [9]. And the slightly decreased Mn─O and Mn─Mn bond lengths for A‐LRMO cathode compared to that of B‐LRMO cathode at initial state could be ascribed to S, Zr co‐doping effect (Figures S32 and S33 and Table S6), which help balance lattice stability and Li^+^ ions migration. Noticeably, for B‐LRMO cathode, the Mn─Mn bond contracts from 3.01 to 2.88 Å, owing to the formation of Li/O vacancies and the lattice transformation from layered structures to spinel structures, which consist with XANES results. As for A‐LRMO cathode, no obvious variation could be seen after cycling (from 2.97 to 2.97 Å), demonstrating its robust lattice and enhanced cathodic compatibility during insertion/extraction of Li^+^ ions. And steady chemical environment of Mn element within A‐LRMO cathode accompanied with evident variation of that for B‐LRMO cathode during cycling could also be detected after the wavelet‐transform of k3‐weighted EXAFS signals (Figure 3j–m), and the same conclusion could be easily draw from this perspective. For In K‐edge XANES spectra, the B‐LRMO‐based composite cathode exhibits a distinct tendency toward oxidation by oxygen radicals after cycling, with its peak profile shifting toward that of In_2_O_3_ sample [10], whereas no significant change is observed for the catholyte cycled A‐LRMO‐based catholyte (Figure 3n). In contrast, no significant signal offset occurs for A‐LRMO cathode, which manifests the preferable cathodic compatibility during cycling (Figure 3o). For Fourier‐transformed EXAFS spectra of In K‐edge (Figure 3p,q), the evident peaks representing typical In─Cl shell in LIC locate at around 1.9 Å for both B‐LRMO and A‐LRMO cathodes before cycling. After cycling, the average bond length for LIC within B‐LRMO composite cathode decreases to around 1.8 Å, which is closer to that of typical In─O (around 1.6 Å) in In_2_O_3_, indicating that severe corrosion effect of LIC by oxygen radicals released from LRMO lattice leads to the formation of In─O coordination as XPS results. On the contrary, the signal for LIC within A‐LRMO cathode after cycling exhibits no apparent variation at first shell, which demonstrates the less oxygen radicals released from LRMO lattice during cycling.

**FIGURE 4:**
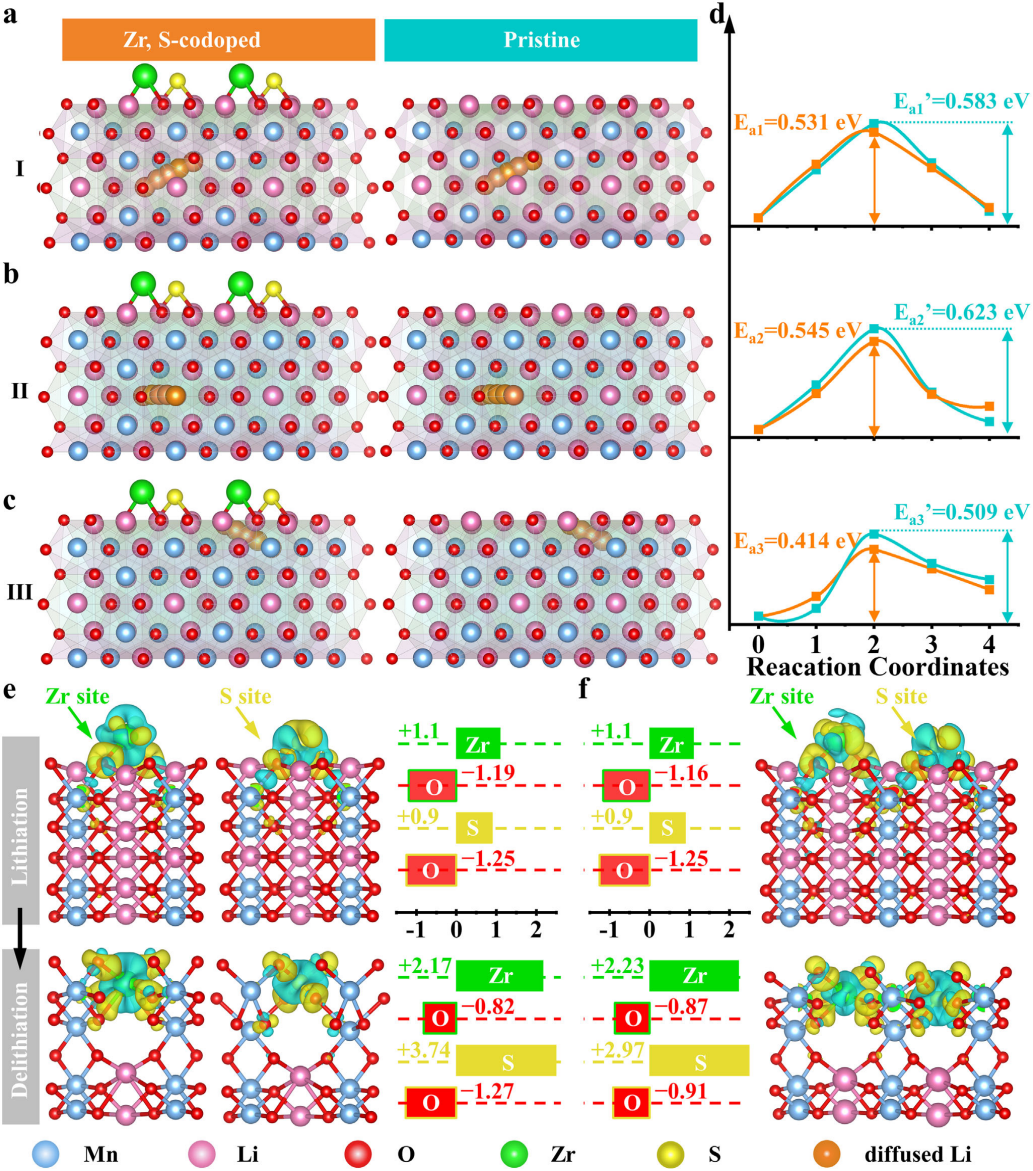
The DFT calculations for Li^+^ ions diffusion in the (a,b) bulk and (c) near surface of Zr, S co‐doping LRMO and pristine LRMO structures, accompanied with corresponding (d) energy barriers. The charge density difference of (e) Zr‐doping, S‐doping and (f) co‐doping LRMO lattice at lithiation and delithiation states, accompanied with corresponding calculated Bader charge of Zr, S atoms, and O atoms nearby.

To evaluate the influence of surface functionalization on Li^+^ mobility, we investigated the diffusion barriers of Li^+^ across three representative paths (I–III) on both Zr, S surface‐functionalized and pristine LRMO (Figure 4a–c; Figures S34–S36). In the functionalized lattice, Zr and S atoms were anchored at the surface. The atomic configurations along the diffusion paths clearly illustrate the altered energy landscape due to the presence of surface species. The corresponding energy profiles (Figure 4d) demonstrate a consistent reduction in activation energy for the surface‐functionalized model 0.531 vs. 0.583 eV (path I), 0.545 vs. 0.623 eV (path II), and 0.414 vs. 0.509 eV (path III) relative to the pristine counterpart. These findings confirm that Zr and S surface functionalization effectively lowers the Li^+^ diffusion barriers, thereby enhancing ionic conductivity.

To understand the mechanism underlying the improved structural stability in Zr, S co‐functionalized LRMO, the charge density difference maps and Bader charges were analyzed (Figure 4e,f). In the pristine LRMO surface, charge accumulation between adjacent surface oxygen atoms during delithiation drives spontaneous formation of unstable O─O dimers. However, Figure 4e,f reveals that upon Zr and S surface functionalization, whether co‐doping or not, electrons accumulate predominantly around the introduced Zr and S atoms rather than between the surface O atoms. Simultaneously, electron depletion regions clearly appear around the adjacent oxygen atoms, effectively delocalizing charge and suppressing localized O─O bond formation [27].

Quantitative analysis via Bader charges (Figure 4f) further supports this stabilizing effect. Initially at lithiation state, the Bader charges remain similar for single Zr, S, and Zr/S co‐functionalized models, with moderate positive charges on Zr (+1.1 |e|) and S (+0.9 |e|) sites, whereas O atoms exhibit relatively high negative charges (−1.16–−1.25 |e|). Upon delithiation, the increased Bader charges could be observed for Zr (+2.17 |e|) and S (+3.74 |e|) elements in the singly doped system, which effectively avoid the over oxidation of lattice O and suppress the formation of O radicals. As for co‐doping of Zr and S elements, both dopant elements (+2.23 |e| for Zr and +2.97 |e| for S) and lattice oxygen (−0.87 |e| for O around Zr and −0.91 |e| for O around S) atoms exhibit more uniform increases in Bader charges, and such charge redistribution capability induced by a strong polyanionic‐type interaction, effectively preventing localized electron accumulation between oxygens and suppressing the formation of unstable O─O dimers [25, 26]. Collectively, these results confirm that Zr and S surface functionalization stabilizes the surface by altering the local electronic environment and inhibiting structural degradation during electrochemical cycling, and the fast kinetics for Li^+^ ions diffusion are also evidenced by DFT calculation, which fundamentally ensures long cycling life and exceptional rate capability of A‐LRMO in ASSLBs.

### 2.4 ASSLBs Performance Validation

**FIGURE 5:**
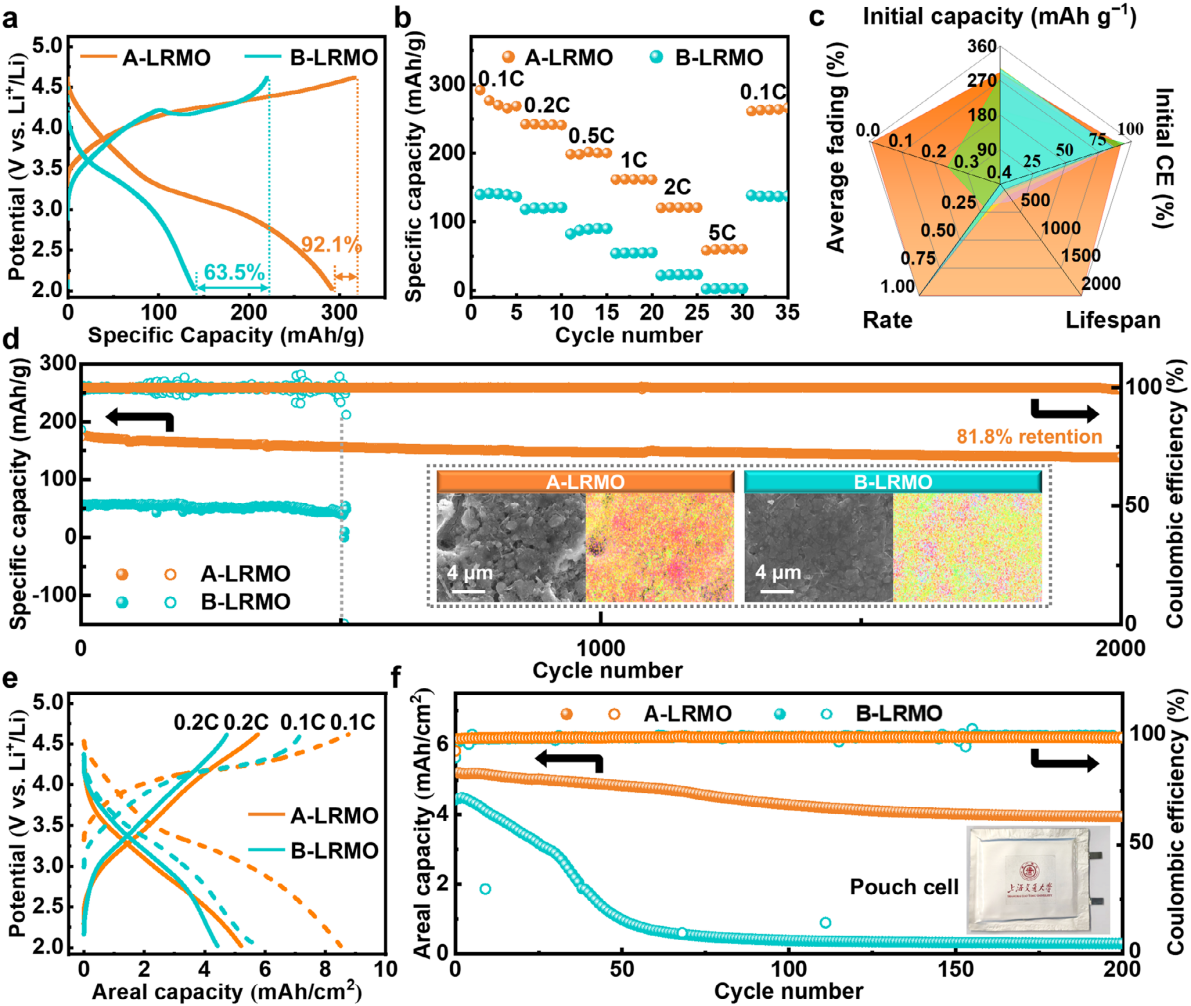
(a) The initial charge–discharge curves at 0.1C (1C = 350 mA g^−1^), (b) rate performances and (d) long‐term cycling stabilities at 1C of A‐LRMO‐LIC VGCF/LPSC/Li‐In and B‐LRMO‐LIC‐VGCF/LPSC/Li‐In ASSLBs at RT. (c) Performance comparison between previous representative works [55, 56, 57, 58, 59, 60] and ours (orange) with comprehensive consideration of initial discharge capacity, cycling life, rate, capacity retention, etc. (d) Long‐term cycling stabilities at 1C (1C = 350 mA g^−1^) of A‐LRMO‐LIC VGCF/LPSC/Li‐In and B‐LRMO‐LIC‐VGCF/LPSC/Li‐In ASSLBs at RT. (e) The charge‐discharge curves and (f) long‐term cycling stabilities at 0.2C (1C = 350 mA g^−1^) of A‐LRMO‐LIC VGCF/LPSC/Li‐In and B‐LRMO‐LIC‐VGCF/LPSC/Li‐In ASSLBs at RT based on pouch cells.

To further validate the practical application value of this modification strategy, mould cells and pouch cells based on A‐LRMO and B‐LRMO·cathodes were assembled and subjected to performance testing. As shown in Figure 5a, the ASSLBs employing A‐LRMO cathode demonstrates an initial discharge capacity of 292.04 mAh/g with Coulombic efficiency (CE) of 92.1%, while the B‐LRMO counterpart exhibits 140.0 mAh/g with CE of 63.5%, which clearly indicates that irreversible reactions (such as oxygen loss and interfacial decomposition) are suppressed. And the detailed dQ/dV curves in Figure S37 confirm that the highly reversible redox of anion ions and rapid Li^+^/e^−^ transport kinetics of A‐LRMO with gradient modification become the vital factors for significantly improved battery performance. In terms of rate performance, the A‐LRMO‐based ASSLBs deliver discharge capacities of 242.7, 198.7, 162.1, 120.4, and 58.4 mAh/g at 0.2, 0.5, 1, 2, and 5C, respectively, all surpassing those of the B‐LRMO cathode (139.8, 118.4, 82.5, 54.1, and 22.0 mAh/g). When the rate reverts to 0.1C, the A‐LRMO sample retains a high discharge capacity of 261.5 mAh/g, demonstrating its excellent rate performance, whereas the B‐LRMO cathode only retains 131.8 mAh/g (Figure 5b). Even under high‐temperatures of 60°C, the A‐LRMO cathode exhibits significantly higher discharge capacity than the B‐LRMO cathode (299.8 vs. 175.43 mAh/g) while maintaining superior rate performance (Figure S38). These results demonstrate that the gradient modification effectively suppresses interfacial oxygen radical corrosion. Additionally, as shown in Figure 5c,d and Figures S39 and S40, the A‐LRMO sample maintains 81.8% of discharge capacity (139.0 mAh/g) after 2000 cycles at 1C with comparative stable average discharge capacity and exerts a long lifespan of over 5000 cycles while B‐LRMO sample exhibits a low discharge capacity below 54.6 mAh/g followed by a sharp capacity drop and rapid failure after only 500 cycles, which exhibits comprehensive application advantages over reported works in terms of initial discharge capacity, initial CE, lifespan, and capacity retention (Table S7).

As seen in Figures S41 and S42, the SEM observations for both A‐LRMO and B‐LRMO based composite cathodes present the characteristics of being smooth, uniform and dense, which indicate intimate contact between active materials, LIC and VGCF identified by elemental distributions, ensuring rapid Li^+^/e^−^ pathways within composite cathodes before cycling. The evident porous and cracks could be detected for B‐LRMO based composite cathode after cycling (Figure S43), whereas the morphology of A‐LRMO based composite cathode maintains still with no obvious structural collapse (Figure S44). Correspondingly, the larger bulk resistance and evident interfacial semicircle for the EIS profile of B‐LRMO based ASSLB compared to that of A‐LRMO sample could also be observed (Figure S45), and rapid Li^+^ ionic transfer of the cathodic interface is maintained for the gradient‐modified A‐LRMO cathode with a high diffusion coefficient after long‐term cycling test (Figure S46). The Raman analyses of composite cathodes after cycling reveal that the apparent Mn─O mode appears for B‐LRMO sample, indicating the formation of secondary Li_2_MnO_3_ phase, which leads to voltage decay and structural degradation as mentioned before (Figure S47). In contrast, the A‐LRMO sample maintains a stable lattice structure, showing only A1g and Eg modes representing hexagonal O2‐type layered structure, as well as carbon‐related signals. And the severe oxygen radical corrosion effect on LIC for B‐LRMO during cycling is further confirmed by XPS results with evident In‐O bond formation after cycling, whereas the lattice structure of LIC maintains unchanged for A‐LRMO composite cathode (Figure S48). Through the above analysis of the structure and composition of the cathodic interfaces, the exceptional interfacial stability enabled by gradient‐modified method for A‐LRMO cathode have been comprehensively revealed. Furthermore, pouch cells for ASSLBs were also assembled and tested, employing high areal mass loading of 27.82 mg/cm^2^ to meet commercial application requirements. As shown in Figure 5e, the A‐LRMO‐based pouch cell demonstrates discharge areal capacities of 8.5 and 5.2 mAh/cm^2^ at 0.1C and 0.2C, respectively, outperforming the B‐LRMO counterpart (5.5 and 4.4 mAh/cm^2^, respectively). This indicates superior Li^+^/ e^−^ transport kinetics within A‐LRMO cathode. Therefore, the energy density of the pouch cell employing A‐LRMO is calculated to be 191.51 Wh/kg (Table S8), which exerts evident improvement comparing to that of mould cell (21.15 Wh/kg). Should the issues of lithium metal interface stability and cycle life be further addressed, the energy density of this pouch cell is projected to increase to 420 Wh/kg, demonstrating promising application potential compared to related studies. Additionally, the A‐LRMO pouch cell maintains stable cycling performance at 0.2C, retaining 4.18 mAh/cm^2^ after 200 cycles with average CE consistently above 99.6%. And the average discharge voltage of pouch cell also maintains steady during cycling (Figure S49), which only reduces a little from 2.91 to 2.85 V (vs. Li^+^/Li). In contrast, the B‐LRMO pouch cell exhibits rapid capacity decay and poor CE, reflecting inferior cathodic chemistry/electrochemical compatibility (Figure 5f). As a conclude, the preferable cathodic compatibility accompanied with superior rate performance and long lifespan collectively validate the commercial viability of the gradient‐modified A‐LRMO cathode.

## 3 Conclusions

A novel A‐LRMO cathode material with a gradient‐modified structure was synthesized through a one‐step mechano‐fusion process using Zr(SO_4_)_2_ as the precursor. The combined effects of S/Zr co‐doping and an amorphous Zr(SO_4_)_2_ coating synergistically stabilize the LRMO lattice, suppress oxygen dimerization, and enhance anionic redox reversibility. DFT calculations reveal reduced Li^+^ diffusion barriers (0.531–0.414 eV) and uniform charge redistribution between dopant and lattice oxygen, accounting for improved structural and electrochemical stability. The soft, ion‐conductive coating ensures intimate contact and rapid Li^+^ ionic conductivity, which has been verified by DC polarization, GEIS, CV, and GITT analyses. Operando Raman, XPS, and XANES/EXAFS characterizations further confirm stable interfaces between cathode and solid electrolytes, and suppressed oxygen‐radical degradation. Consequently, the A‐LRMO cathodes delivered a high discharge capacity of 292 mAh/g at 0.1 C, retained 81.8% capacity after 2000 cycles at 1 C, and maintained a lifespan exceeding 5000 cycles. Pouch cells achieved areal capacities up to 8.5 mAh/cm^2^ and 99.6% Coulombic efficiency. This work showcases gradient‐engineered A‐LRMO cathodes for high‐energy, long‐life all‐solid‐state lithium batteries.

## Conflicts of Interest

The authors declare no conflicts of interest.

## Supporting information




**Supporting File**: adma72233‐sup‐0001‐SuppMat pdf.

## Data Availability

The data that support the findings of this study are available from the corresponding author upon reasonable request.
