# Supplementary material for: Gradient‐Modified Li‐Rich Manganese‐Based Oxides Cathodes with Breakthrough of Kinetic Limitation for High‐Performance All‐Solid‐State Lithium Metal Batteries
Source: Adv Mater. 2026 Jan 28;38(13):e21690. doi: 10.1002/adma.202521690 (PMC12957873; doi:10.1002/adma.202521690)
Supplement: Supplementary file 1 — Supporting File: adma72233‐sup‐0001‐SuppMat pdf. [file ADMA-38-e21690-s001.pdf]

Supporting Information

Gradient-Modified Li-rich Manganese-Based Oxides Cathodes with Breakthrough of Kinetic Limitation for High-Performance All-Solid-State Lithium Metal Batteries

Ya Chen§*, Ling Huang*§*, Awais Ghani*§*, Xiaodong Chen*, Xin Gao, Tao Meng, Hongchao Sun, Runjing Xu, Peiyi Ji, Xianghong Deng, Long Bao, Shuang Wang, Nana Wang, Gang Tan*, Guoxiu Wang*, Zhen Huang**

1. Experimental section

1.1 Materials preparation

All the preparation processes were performed in an argon-filled glovebox (O_2< 0.1 ppm, H_2O < 0.1 ppm).

1.1.1 Preparation of Li_6PS_5Cl (LPSC) electrolyte:

The synthesis of LPSC was conducted under strict argon protection due to the material's sensitivity to moisture and oxygen. High-purity reagents (≥99.9%) were prepared in stoichiometric proportions: Li_2S: P_2S_5: LiCl = 5: 1: 2 (molar ratio).

The pre-mixed powders underwent mechanical activation via planetary ball milling (600 rpm, 10 h) using ZrO_2 grinding media within a ZrO_2 container. Subsequent thermal treatment involved controlled annealing at 550°C for 6 hours with a heating ramp of 5°C/min.

Critical operations including weighing, homogenization, milling, annealing, and sample transfer were all performed within an inert gas glovebox to prevent degradation of both the final LPSC material and its intermediate precursors.

1.1.2 Preparation of Li_3InCl_6 (LIC) electrolyte:

The LIC electrolyte was synthesized via a liquid-phase approach: Lithium chloride (LiCl, 99.9%) and indium chloride (InCl3, 99.99%) were proportionally weighed according to stoichiometric ratios and dissolved in deionized water under ambient conditions. The resulting solution was subjected to natural air-drying to yield a precursor hydrate, which subsequently underwent vacuum heat-treatment at 200°C for 4 hours to obtain the final LIC electrolyte product.

1.1.3 Preparation of A-$Li_{1.2}Mn_{0.54}Co_{0.13}Ni_{0.13}O_2$ (A-LRMO) and B-LRMO composite cathodes:

The A-LRMO cathodes were fabricated via a solid-phase approach by combining pristine LRMO (B-LRMO) with 20 wt% $Zr(SO4)_2$ in a zirconia pot at a 1:10 powder-to-balls mass ratio, followed by 20-hour ball milling at 600 rpm to produce the A-LRMO precursor. This material was then blended with LIC electrolyte and VGCF carbon additive in a 60:40:5 mass ratio, manually mixed for 10 minutes using mortar and pestle to form the final composite cathode. An identical procedure was employed for the B-LRMO counterpart, substituting B-LRMO as the active material.

1.2 Cells assembly

All assembly processes were performed in an argon-filled glovebox (O_2 < 0.1 ppm, H_2O < 0.1 ppm) to avoid side reactions between materials and air.

1.2.1 Assembly of steel/composite/steel ion-blocking cells:

For steel/composite A-LRMO cathode/steel ion-blocking cell, the assembly process was as follows: 100 mg of composite A-LRMO cathode was added to a mold with a diameter of 10 mm and pressed at 20 MPa for 10 minutes to form firm composite cathode layer. Subsequently, the stainless steel discs were attached on both sides of composite cathode layer and pressed at 300 MPa for 10 minutes to achieve intimate contact. By that steel/composite A-LRMO cathode/steel ion-blocking cell was assembled. Likewise, the steel/composite B-LRMO cathode/steel ion-blocking cell was prepared using the same method as above, but with composite B-LRMO cathode as the interlayer.

1.2.2 Assembly of Li-In/LIC/composite/LIC/Li-In electron-blocking cells:

For Li-In/LIC/composite A-LRMO cathode/LIC/Li-In electron-blocking cell, the assembly process was as follows: 100 mg of composite A-LRMO cathode was added to a mold with a diameter of 10 mm and pressed at 20 MPa for 10 minutes to form firm composite cathode layer. Subsequently, the 100 mg of LIC electrolyte were evenly spread on both sides of the composite cathode layer, respectively, and pressed at 300 MPa for 10 minutes to achieve intimate contact. Finally, 200 mg Li-In anode (with a mass ratio of Li to In of 1:30) was uniformly pressed on the both sides, followed by pressing at 30 MPa for 1 minute, By that, the Li-In/LIC/composite A-LRMO cathode/LIC/Li-In electron-blocking cell was assembled. Likewise, the Li-In/LIC/composite B-LRMO cathode/LIC/Li-In electron-blocking cell and Li-In/LIC/LIC/LIC/Li-In electron-blocking cell were prepared using the same method as above, but with composite B-LRMO cathode and LIC as the interlayers, respectively.

1.2.3 Assembly of A-LRMO-LIC-VGCF/LPSC/Li-In and B-LRMO-LIC-VGCF/LPSC/Li-In ASSLBs:

100 mg of LPSC was added to a mold with a diameter of 10 mm and pressed at 10 MPa for 5 minutes to form the firm SSE layer. Subsequently, 12 mg A-LRMO composite cathode was evenly spread on one side of the SSE and pressed at 300 MPa for 10 minutes to achieve intimate contact. Finally, 200 mg Li-In anode (with a mass ratio of Li to In of 1:30) was uniformly pressed on the other side of the SSE, followed by pressing at 30 MPa for 1 minute, By that, A-LRMO-LIC-VGCF/LPSC/Li-In and B-LRMO-LIC-VGCF/LPSC/Li-In ASSLBs were assembled with the areal mass loading of around 10.70 mg/cm^2.

1.2.4 Assembly of A-LRMO-LIC-VGCF/LPSC/Li-In and B-LRMO-LIC-VGCF/LPSC/Li-In pouch cells:

To fabricate a dry film of the cathode, the composite cathode was mixed with 1.5 wt% polytetrafluoroethylene (PTFE) in a heated mortar. After 3 min of mixing and shearing, a single flake was formed.[1]

The 60 mm × 46 mm pouch cell with an area mass loading of 27.82 mg/cm^2 was produced.[2] And the sheets were cut by a punching machine. The dimensions of the anode, cathode, and LPSC sheet were 58 mm × 44 mm, 54 mm × 40 mm, and 60 mm × 46 mm, respectively. All electrodes and LPSC sheets were stacked and packaged in a laminated bag. After the vacuum was applied to the laminate bag and sealed, the battery was pressurized for 10 m Electrodes and LPSC in by a Warm Isostatic Press (WIP400, Sichuan Lineng Ultra High Voltage Equipment Co., Ltd) at a pressure of 300 MPa. Then, the battery was taken out of the laminate bag and the Al and Ni terminals of the cathode and anode, respectively, were welded using an ultrasonic welder. The battery was placed in a laminated bag and vacuum-sealed again. To be mentioned. most of the assembly processes were carried out in the argon atmosphere owing to the instability of Electrodes and LPSC against moisture and oxygen.

1.3 Materials characterizations

1.3.1 X-ray diffraction (XRD):

X-ray diffraction (XRD) analysis was performed using a Rigaku Utima IV diffractometer equipped with Cu Kα radiation, scanning a 2θ range of 10-80°. All samples were encapsulated in Kapton films to prevent exposure to atmospheric oxygen and moisture, thereby minimizing potential side reactions.

1.3.2 X-ray photoelectron spectroscopy (XPS):

X-ray photoelectron spectroscopy (XPS) analysis was conducted using a Thermo Scientific ESCALAB 250xi spectrometer. The system employed monochromatic Al Kα excitation (3000 eV) with an X-ray source operating at 50 W power and 15 kV beam voltage, featuring a focused beam diameter of 900 μm. For depth profiling, surface cleaning was performed using low-energy Ar+ ion sputtering (0.5 kV) to eliminate potential surface interference. The analysis utilized a rasterized scanning area of 6×6 mm^2. All samples were handled under inert conditions, being transferred to the analysis chamber within an argon-filled glovebox to prevent atmospheric contamination.

1.3.3 Characterization of Materials Morphology

The microstructural characterization was performed using a combination of advanced electron microscopy techniques: field-emission scanning electron microscopy (SEM, Zeiss Gemini 300), high-angle annular dark-field scanning transmission electron microscopy (HAADF-STEM, Thermo Themis Z), and aberration-corrected transmission electron microscopy (AC-TEM, Hitachi HF5000). These analytical methods enabled comprehensive examination of sample morphology at multiple scales. Complementary energy-dispersive X-ray spectroscopy (EDS) was employed for spatially resolved elemental distribution mapping, providing chemical composition information correlated with the observed microstructures.

1.3.4 Raman Spectroscopy

Raman (Thermo Fischer DXR) spectroscopy was employed to determine the structure of the samples with the wavenumber ranging from 100 to 2500 cm^{-1}.

1.3.5 X-ray Absorption Near-edge Structure (XANES) and Fourier-transformed extended X-ray absorption fine structure (EXAFS)

X-ray absorption spectra were carried out at ID26 beamline of European Synchrotron Radiation Facility (ESRF at France) with beam size at 1 mm×1 mm. and the data were used to further characterize changes in molecular structure and atomic valence states of the cathodes and solid electrolytes before and after cycling.

1.3.6 Operando Raman spectroscopic measurements

Operando Raman spectroscopic measurements were performed using a Renishaw inVia Qontor spectrometer, covering a spectral range of 100-800 cm^{-1}. The electrochemical cells for operando analysis were securely assembled within customized test fixtures and subjected to charge-discharge cycling at 0.1C rate (where 1C corresponds to 350 $mA \cdot g^{-1}$ for LRMO cathodes). It should be noted that the optimal measurement positions for LPSC signal detection were determined through preliminary screening procedures, ensuring signal intensity matched that of pristine LPSC samples. After identifying these target regions, the exact measurement coordinates were maintained throughout all subsequent in-situ Raman experiments to ensure data consistency.

1.3.7 Four-point probe measurement

The electronic conductivity values were measured at different pressure within the range from 12 MPa to 28 MPa by a semiconductor powder resistivity tester (ST2722).

1.4 Electrochemical measurement

1.4.1 Electrochemical impedance spectroscopy (EIS):

EIS measurements were conducted at frequencies from 1 MHz to 0.1 Hz with the AC amplitude of 10 mV with an Autolab electrochemical workstation (Autolab PGSTAT

302N).

1.4.2 Cycling performance:

Galvanostatic polarization and cycling measurements were conducted using a Neware battery test system (China) and a multichannel battery testing system (LAND CT2001A). ASSLBs with LRMO cathodes were cycled within voltage range of 2.02-4.62 V (vs. Li^+/Li).

1.4.3 Cyclic Voltammetry (CV)

CV curves within voltage ranges of 2.02-4.62 V (vs. Li^+/Li) for ASSLBs assembled with A-LRMO and B-LRMO cathodes at scan rates within the range from 0.05-1.05 mV/s were obtained with an Autolab electrochemical workstation (Autolab PGSTAT 302N).

1.4.4 EIS tests at high cut off voltage

The ASSLBs employing A-LRMO and B-LRMO cathode materials were initially charged to 4.62 V vs. Li^+/Li. For potential-constant mode, the ASSLBs were held at this potential, and for open-circuit voltage mode, the potential was not held. Finally, the EIS measurements were conducted every 30 minutes during this period.

1.4.5 Direct current polarizations (DC)

The DC tests were conducted with an Autolab electrochemical workstation (Autolab PGSTAT 302N). For electronic conductivities tests, the voltage of 500 mV was applied on steel/composite/steel ion-blocking cells. As for Li^+ ionic conductivities, the voltage of 200 mV was applied on Li-In/LIC/composite/LIC/Li-In electron-blocking cells.

1.5 DFT calculation details

Density-functional-theory (DFT) calculations were carried out with the Vienna Ab

initio Simulation Package VASP.[3] Ion–electron interactions were treated with the projector-augmented-wave method, and exchange–correlation effects were described by the Perdew–Burke–Ernzerhof form of the generalized-gradient approximation (GGA).[4] A kinetic energy cutoff of 500 eV was selected based on systematic convergence tests and applied to all subsequent calculations. Geometry optimizations were performed using a Γ-centered Monkhorst–Pack k-point mesh, with convergence criteria set to 10^{-6} eV for total energy and 10^{-2} eV/Å for forces. Electronic-structure calculations to account for the strong electron correction effect in transition metal oxides, electronic structure calculations were performed within the GGA + U framework, employing an effective Hubbard U parameter of 4.5 eV for the Mn 3d electrons. The activation energy barriers for Li-ion migration were evaluated using the climbing image nudged elastic band (CI-NEB) method in both pristine and surface-functionalized structures.[5]

2. List of Supporting Figures and Tables

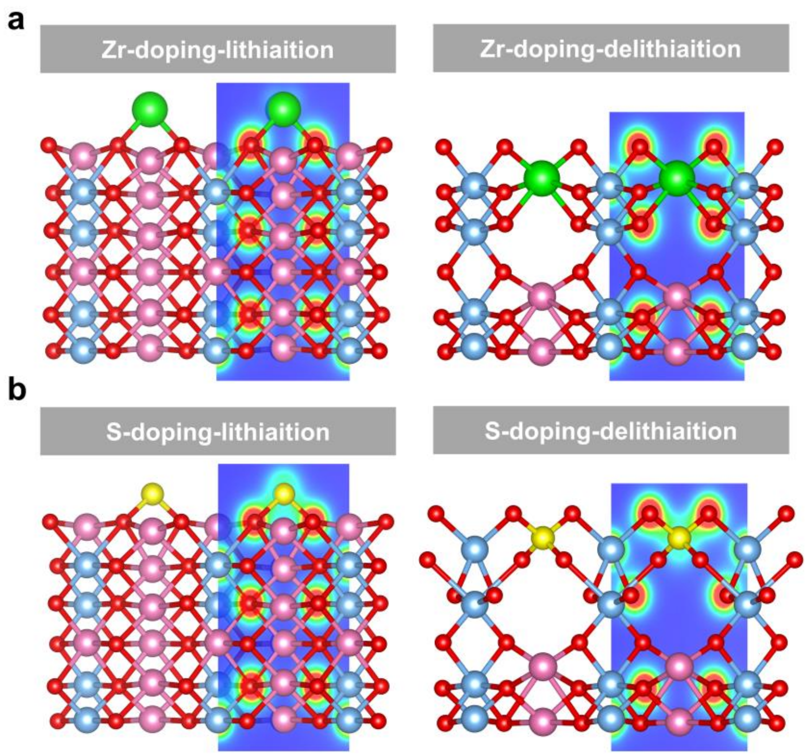

Figure S1. Contour maps of charge density in (a) Zr-doping LRMO, (b) S-doping LRMO at lithiation and delithiation states.

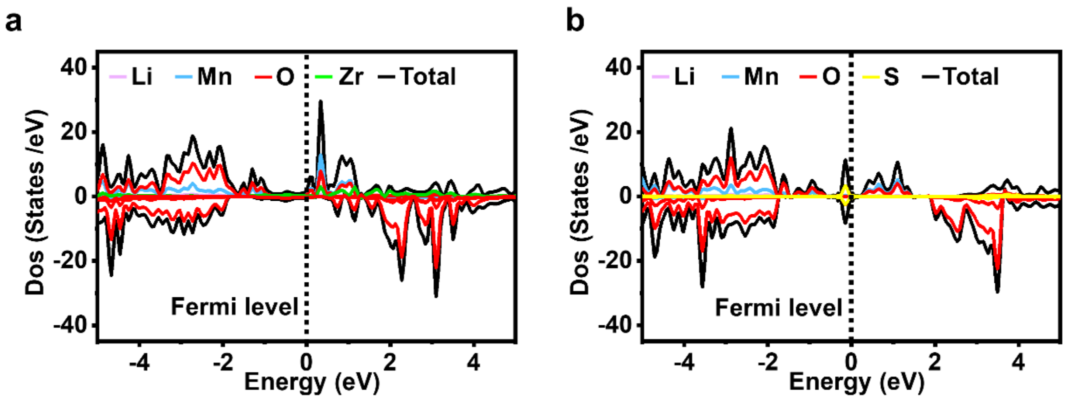

Figure S2. Calculated DOS for (a) Zr-doping LRMO and (b) S-doping LRMO.

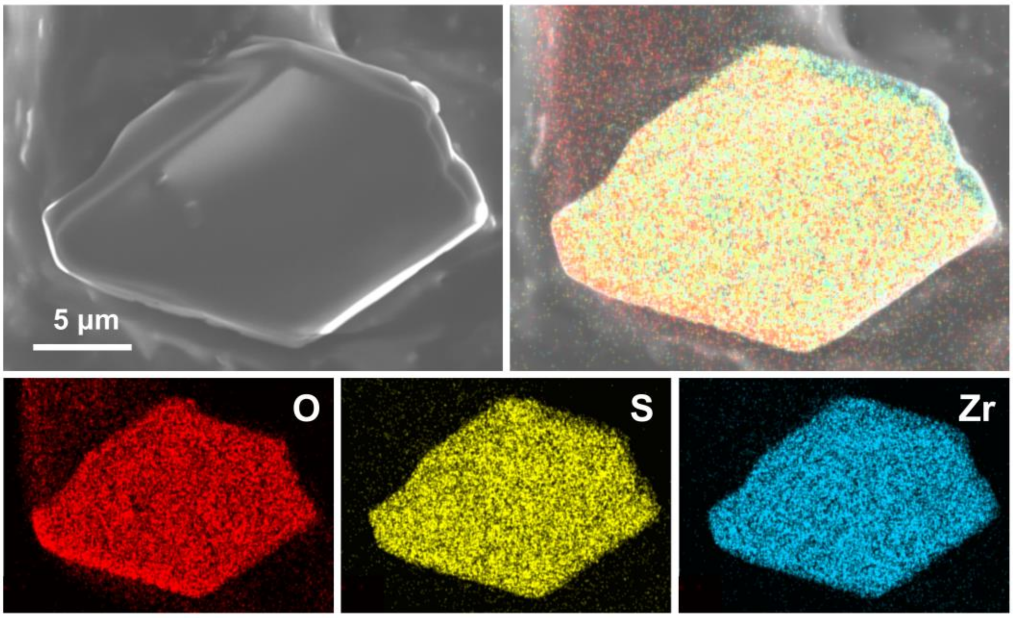

Figure S3. Scanning electron microscope (SEM) observations and energy dispersive spectroscopy (EDS) analyses of $Zr(SO_4)_2$ sample.

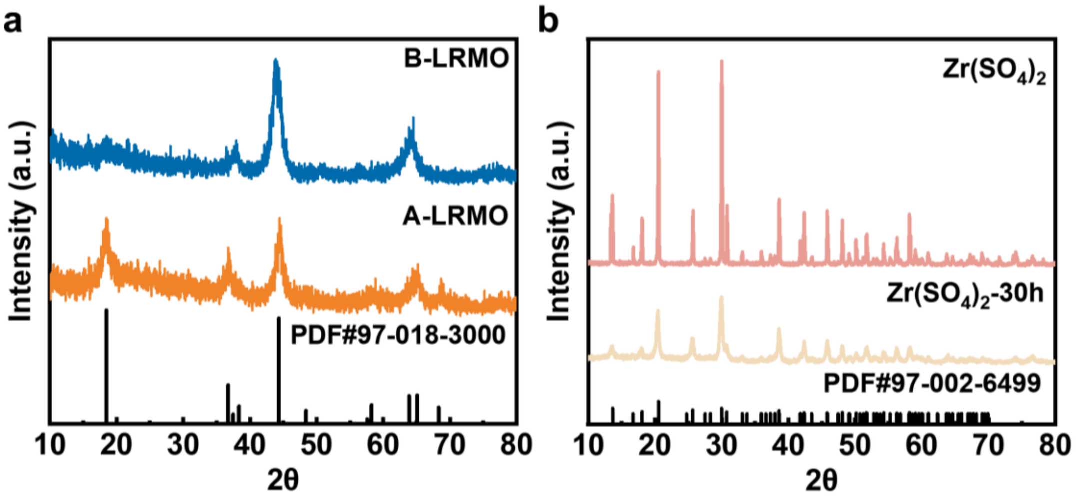

Figure S4. X-ray diffractions (XRD) of (a) A-LRMO and B-LRMO samples. XRD results of $Zr(SO_4)_2$ before and after ball milling for 30 h.

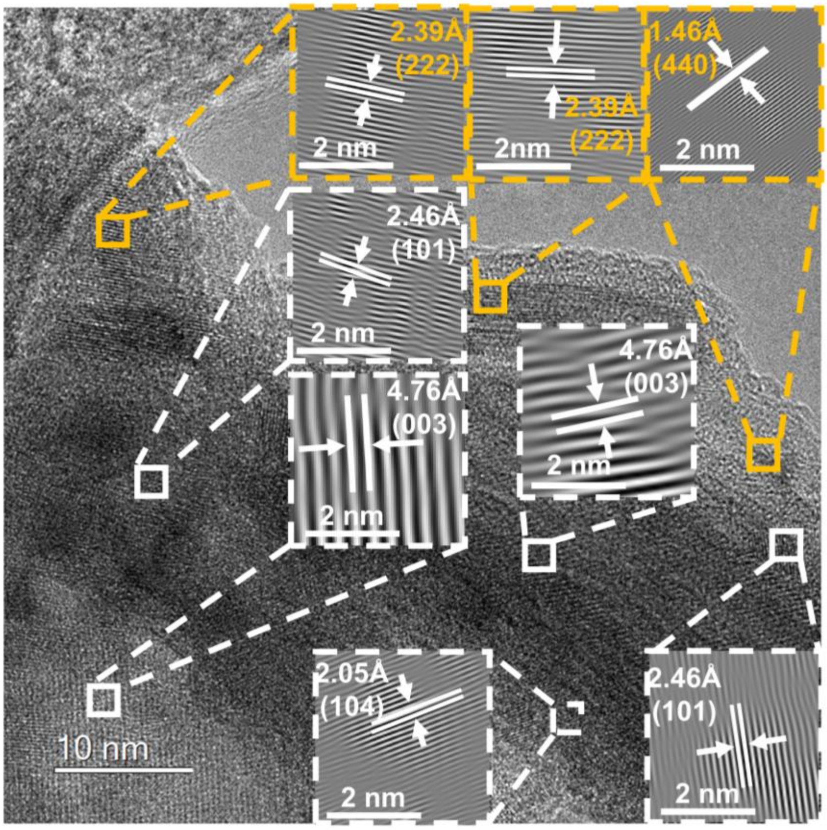

Figure S5. The AC-TEM image of B-LRMO cathode.

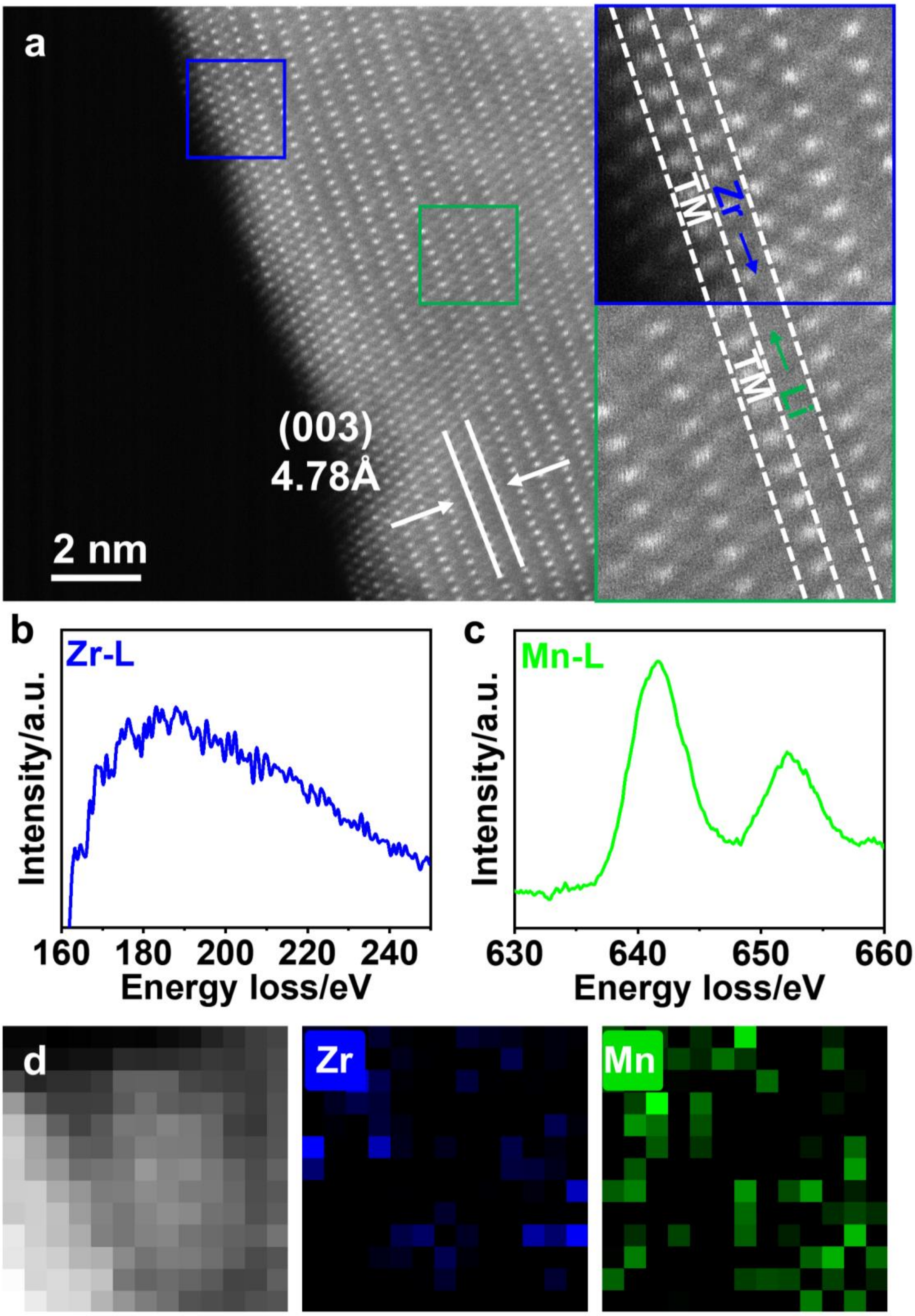

Figure S6. (a) HAADF-STEM images of A-LRMO; (b) EELS line profiling of the Zr L edge of A-LRMO at the surface region; (c) EELS line profiling of the Mn L edge of A-LRMO at the surface region; (d) EELS spectrum and EELS mapping images

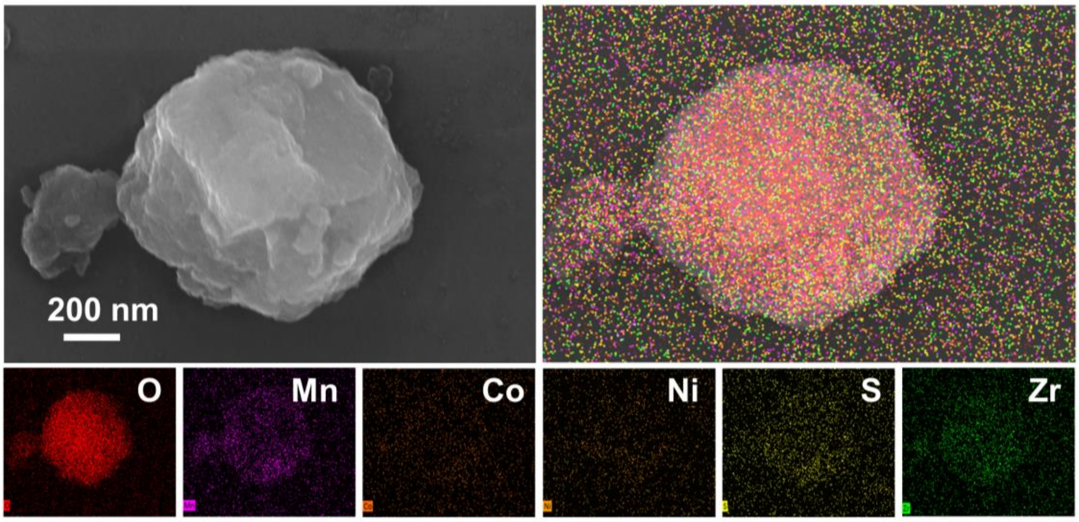

Figure S7. SEM observations and EDS analyses of A-LRMO cathode.

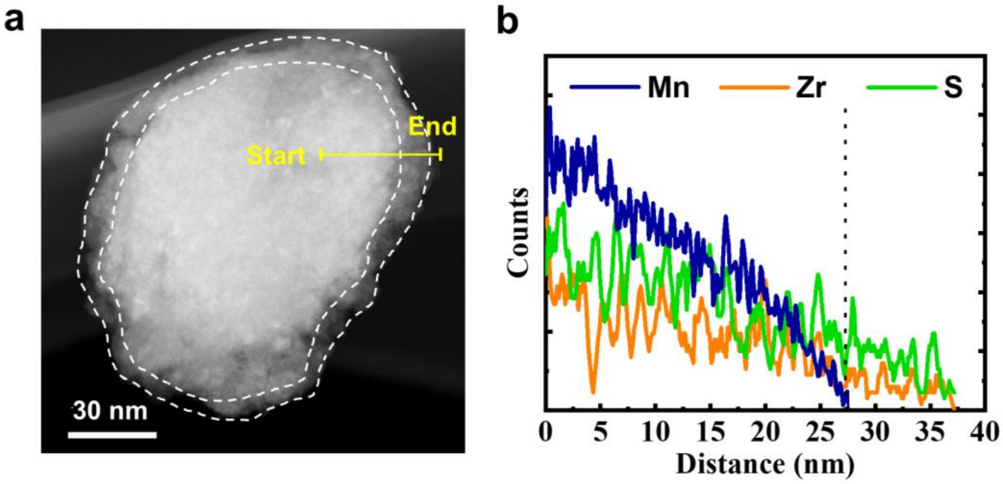

Figure S8. EDS analyses for cathodic interface of A-LRMO sample with linear scanning mode. (a) Full-particle HAADF-STEM panorama of A-LRMO sample accompanied with linear scanning route. (b) The quantitative elemental distributions of S, Zr and Mn along linear scanning route.

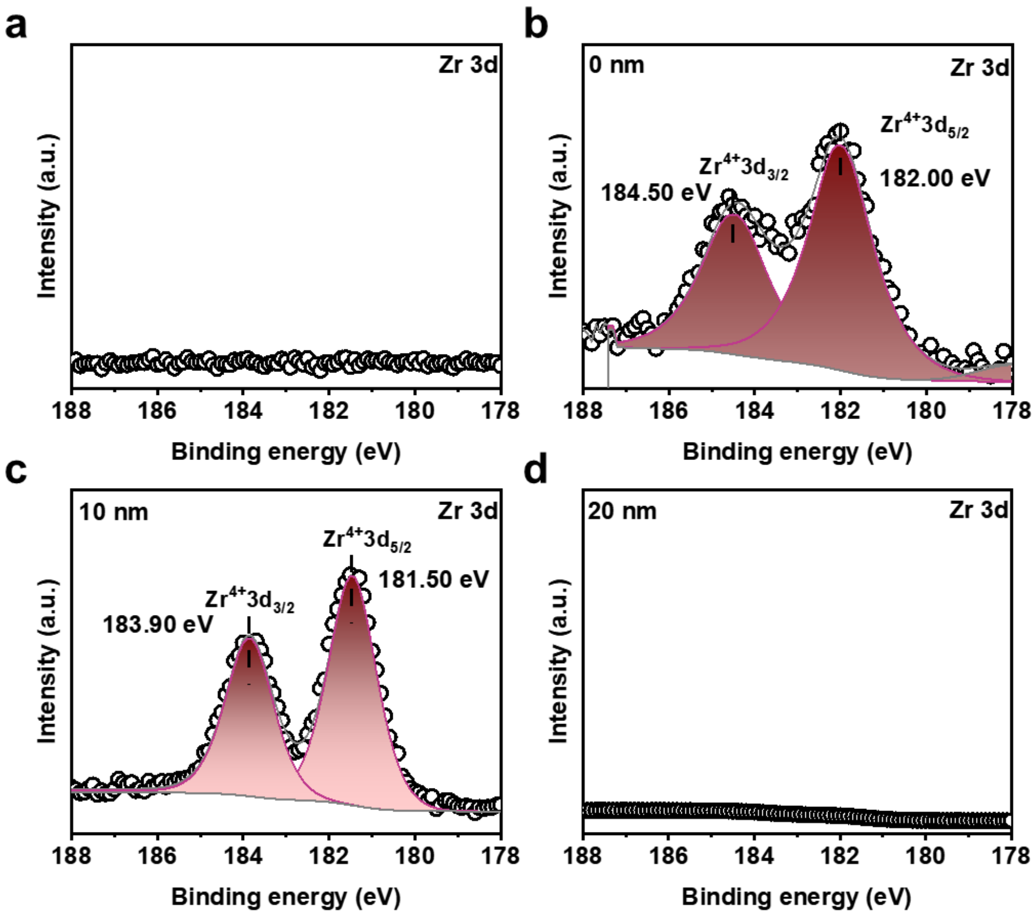

Figure S9. XPS spectra and curve fitting for Zr 3d of (a) B-LRMO and (b, c, d) A-LRMO cathodes at various depths positions after etching.

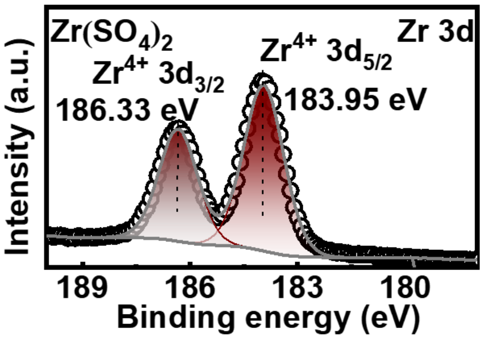

Figure S10. XPS spectra and curve fitting for Zr 3d of $Zr(SO_4)_2$.

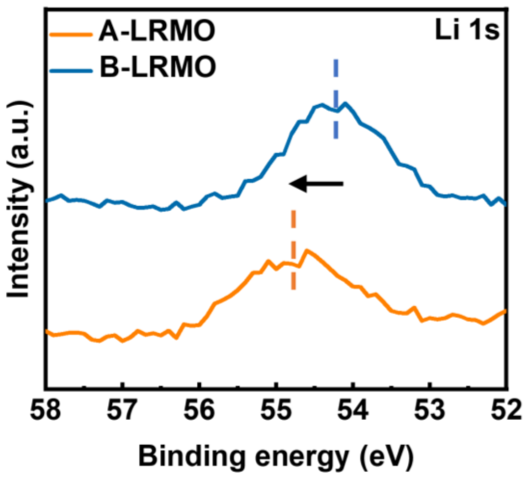

Figure S11. XPS spectra for Li 1s of A-LRMO and B-LRMO cathodes surfaces.

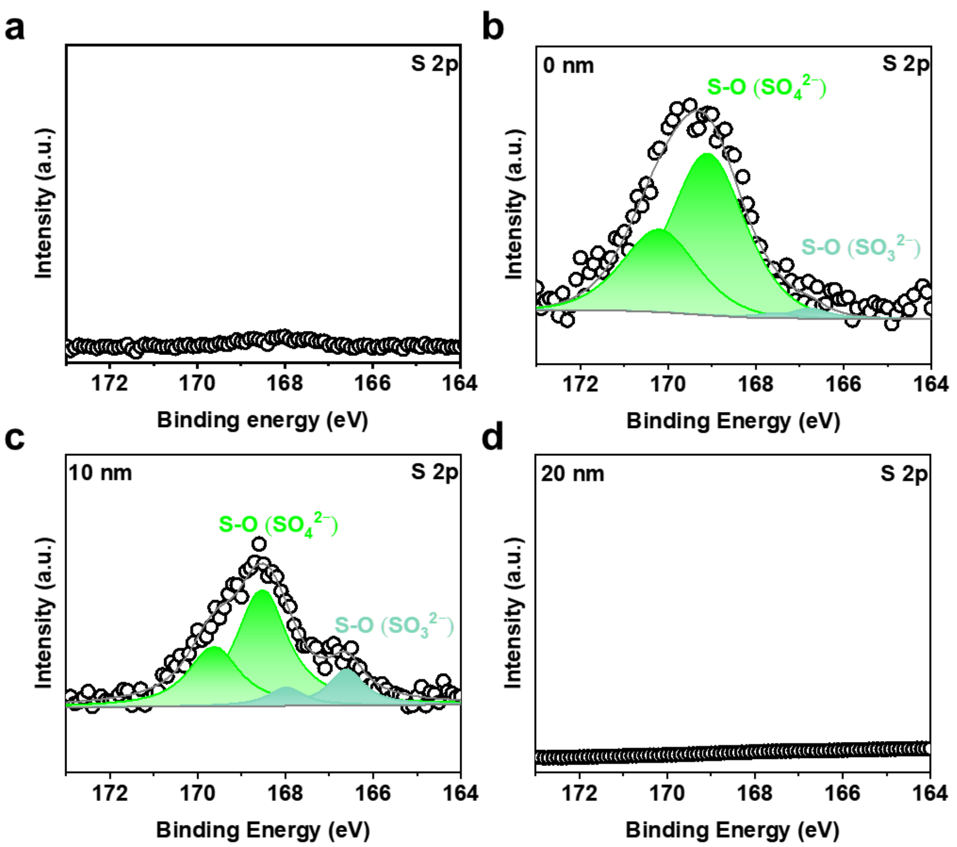

Figure S12. **XPS spectra and curve fitting for S 2p of (a) B-LRMO and (b, c, d) A-LRMO cathodes at various depths positions after etching.**

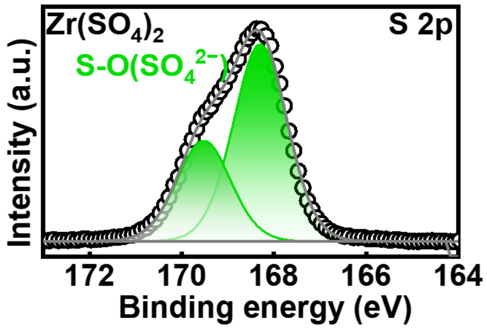

Figure S13. XPS spectra and curve fitting for S 2p of $Zr(SO_4)_2$.

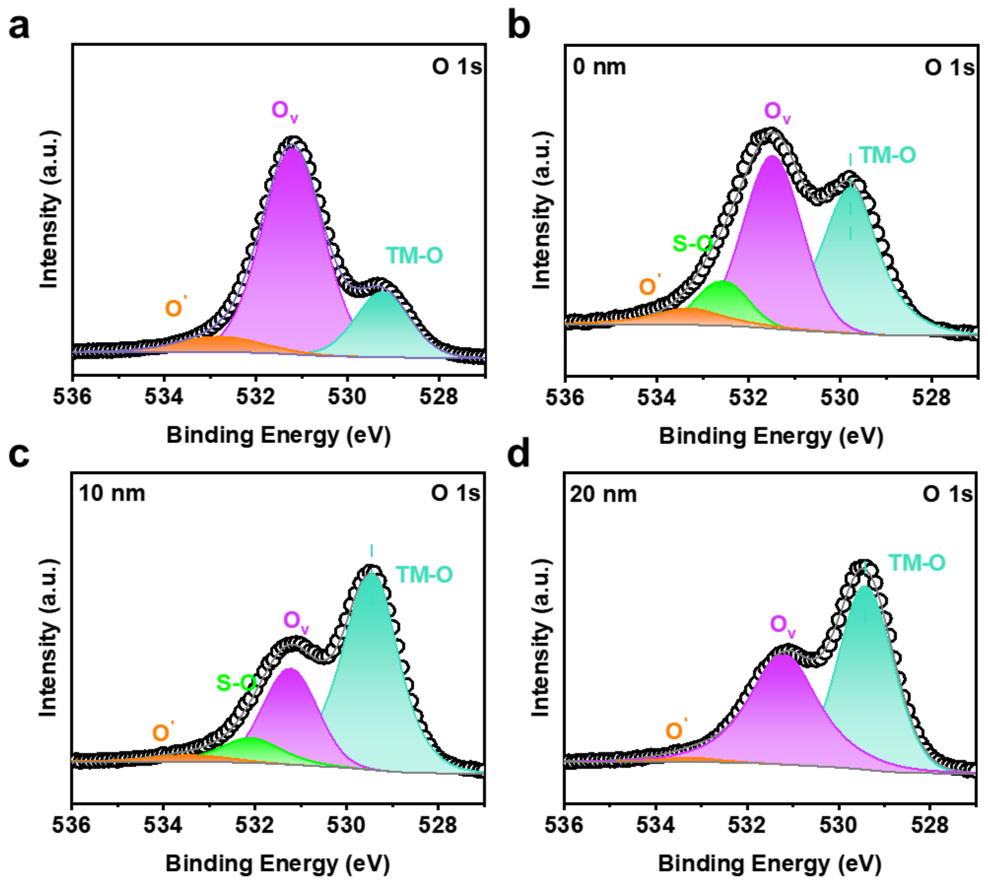

Figure S14. XPS spectra and curve fitting for O 1s of (a) B-LRMO and (b, c, d) A-LRMO cathodes at various depths positions after etching.

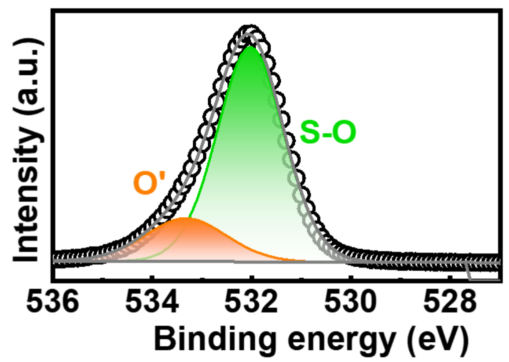

Figure S15. XPS spectra and curve fitting for O 1s of $Zr(SO_4)_2$.

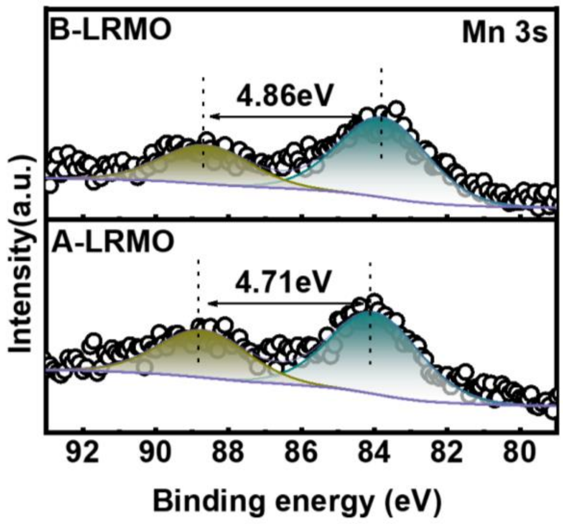

Figure S16. XPS spectra and curve fitting for Mn 3s of A-LRMO and B-LRMO cathodes surfaces.

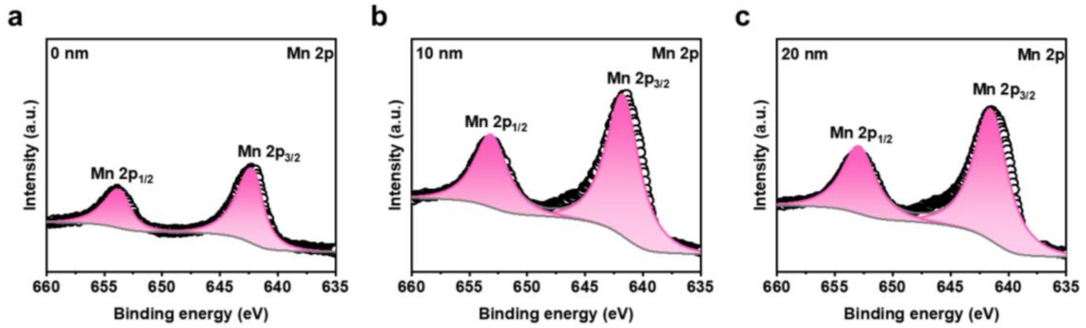

Figure S17. XPS spectra and curve fitting for Mn 2p of A-LRMO at various depths positions after etching.

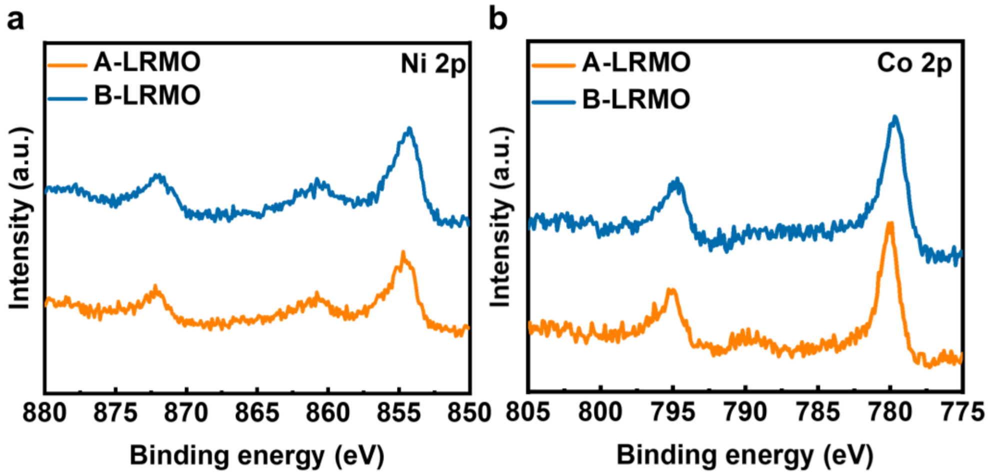

Figure S18. XPS spectra for (a) Ni 2p and (b) Co 2p of A-LRMO and B-LRMO cathodes surface.

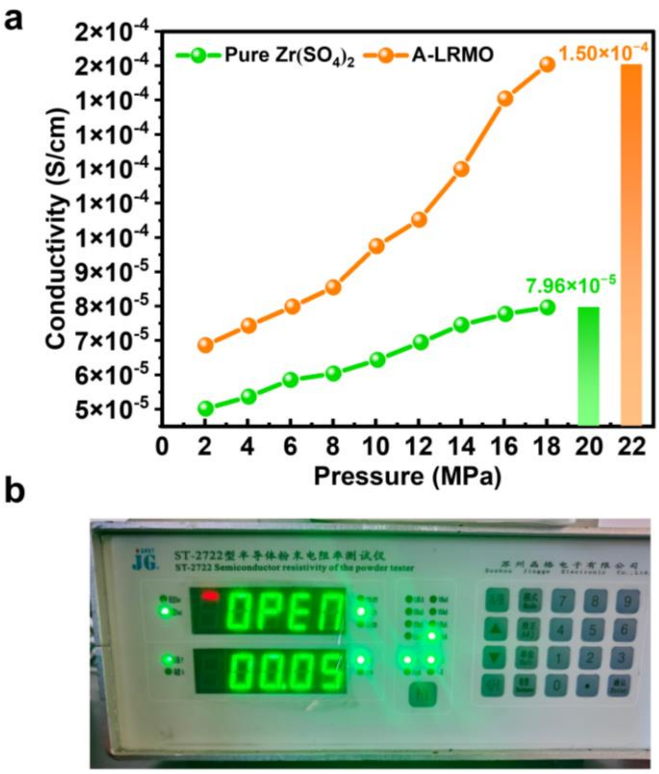

Figure S19. (a) of pure Zr(SO$_4$)$_2$ and A-LRMO samples. (b) The display status on the instrument panel for B-LRMO sample, which is below the detection limit of the semiconductor powder resistivity tester (ST2722) due to its extremely low electronic conductivity.

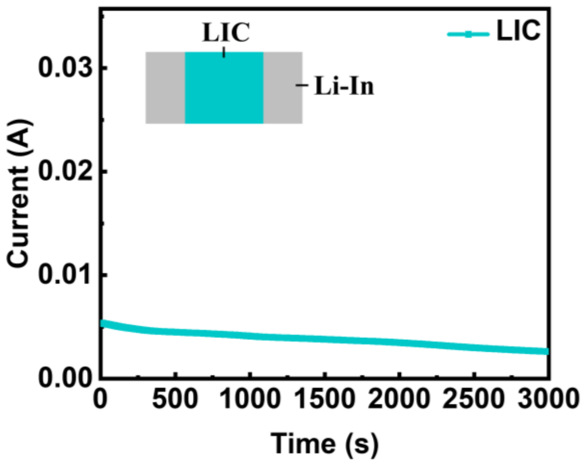

Figure S20. Current–time curve under a polarization of 200 mV in an Li-In/LIC/Li-In cell.

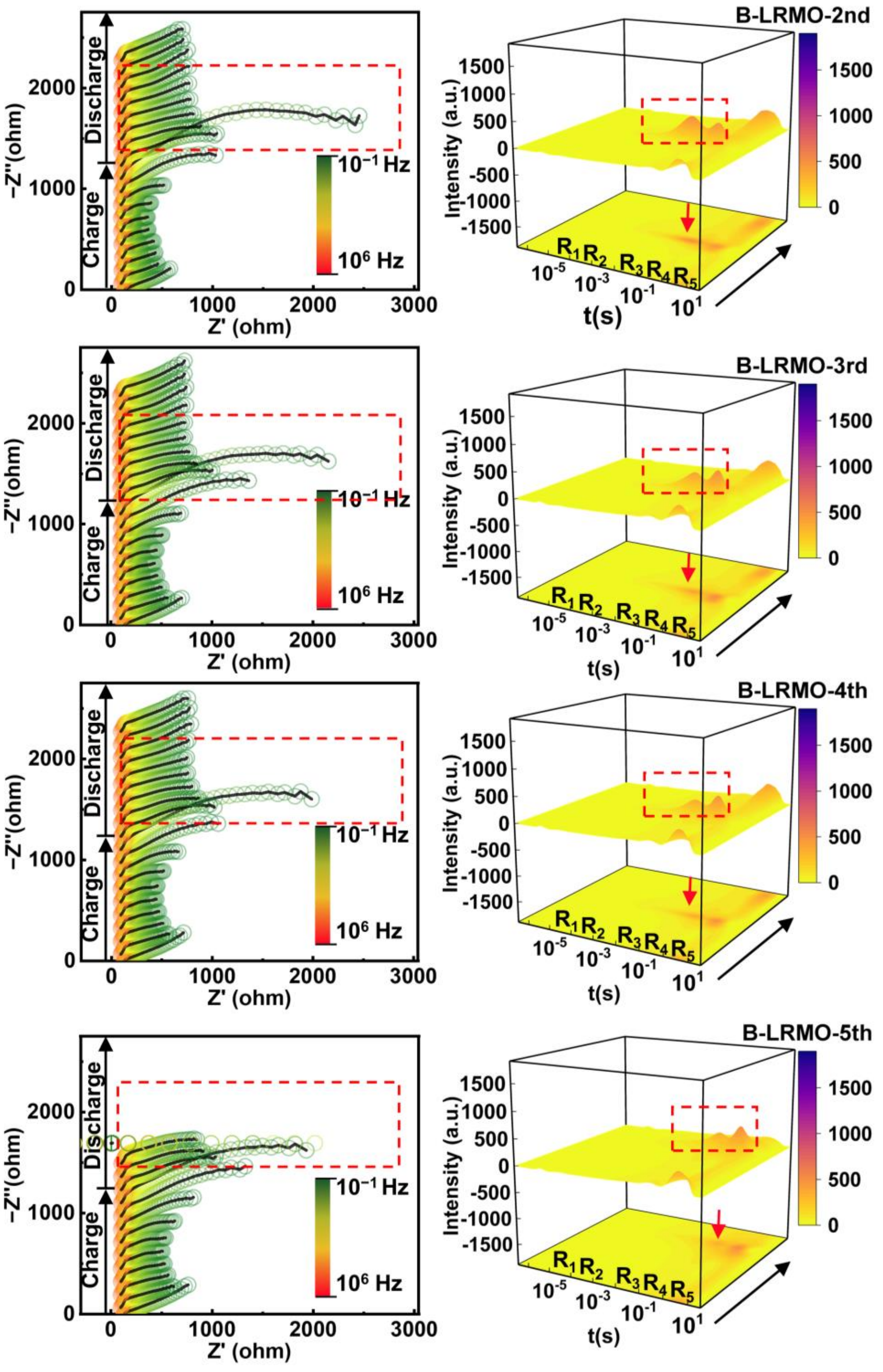

Figure S21. GEIS of B-LRMO-LIC-VGCF/LPSC/Li-In ASSLB during 2nd, 3rd, 4th and 5th cycling accompanied with corresponding contour plots of DRT analyses.

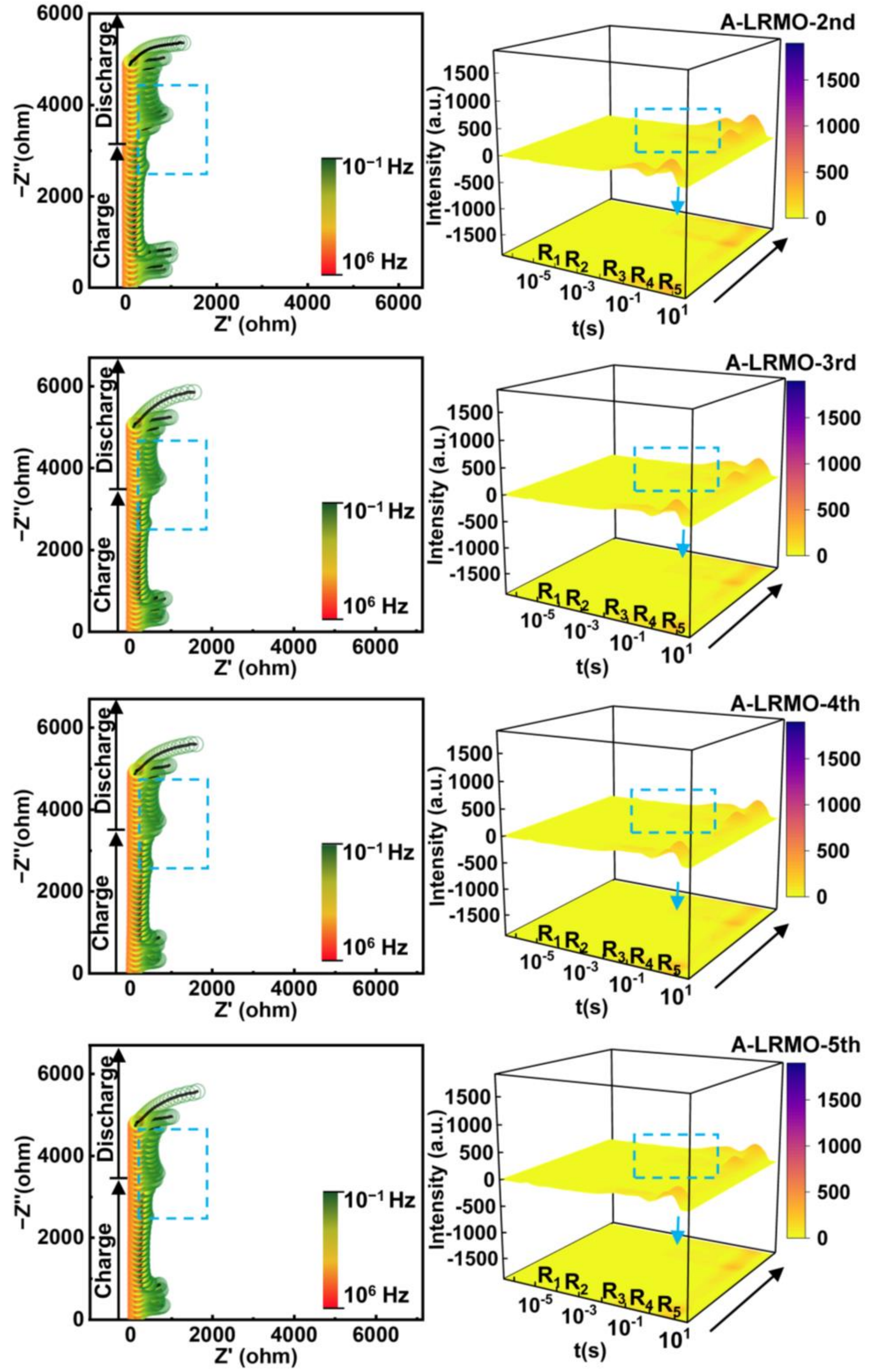

Figure S22. GEIS of A-LRMO-LIC-VGCF/LPSC/Li-In ASSLB during 2nd, 3rd, 4th and 5th cycling accompanied with corresponding contour plots of DRT analyses.

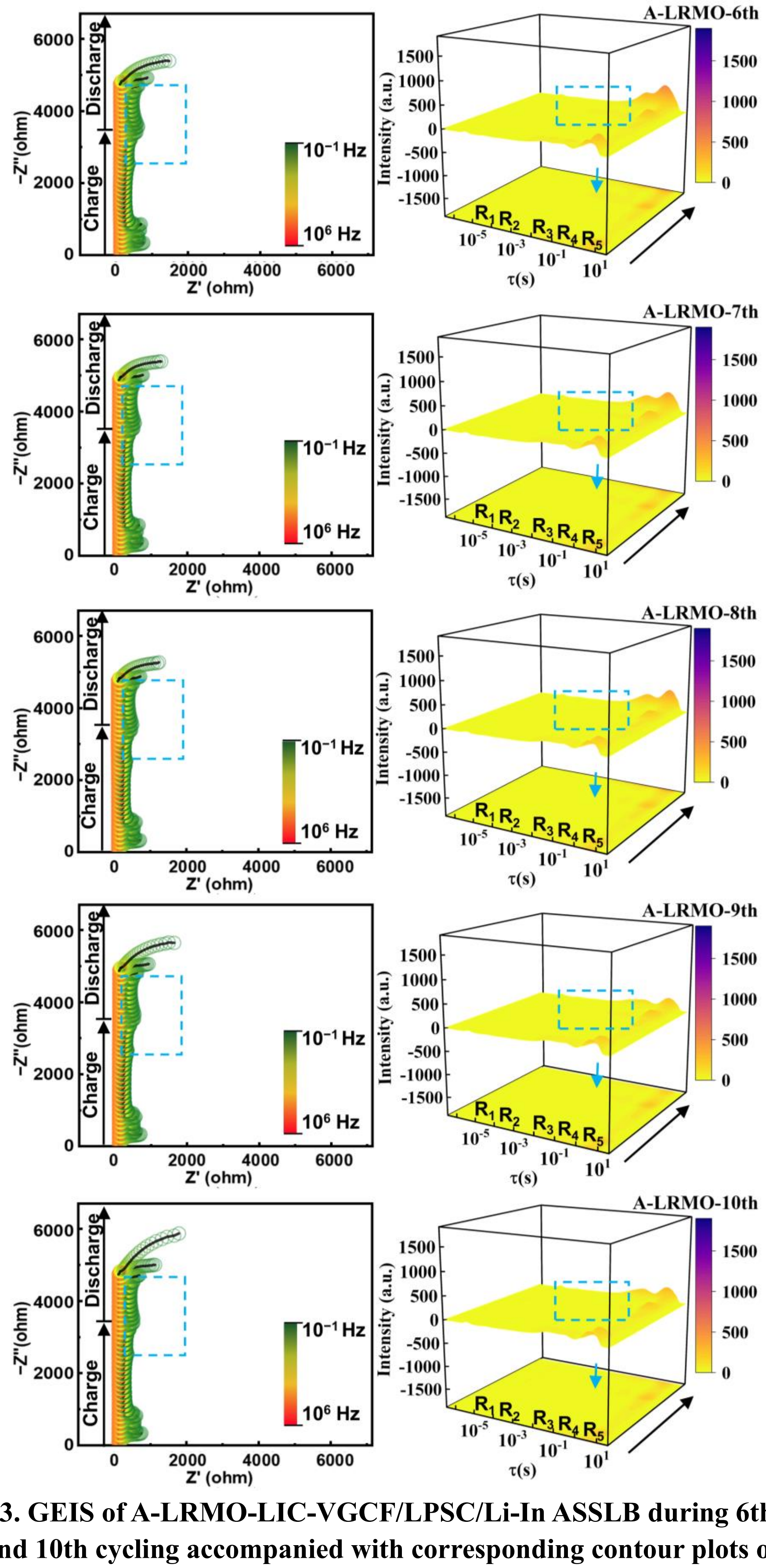

Figure S23. GEIS of A-LRMO-LIC-VGCF/LPSC/Li-In ASSLB during 6th, 7th, 8th, 9th and 10th cycling accompanied with corresponding contour plots of DRT analyses.

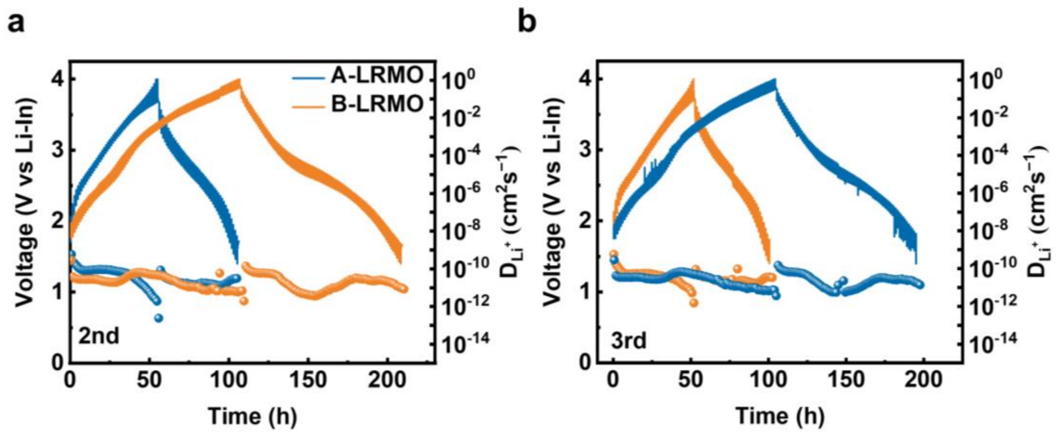

Figure S24. GITT potential curves of B-LRMO-LIC-VGCF/LPSC/Li-In and A-LRMO-LIC-VGCF/LPSC/Li-In ASSLBs during (a) 2nd and (b) 3rd charging and discharging process.

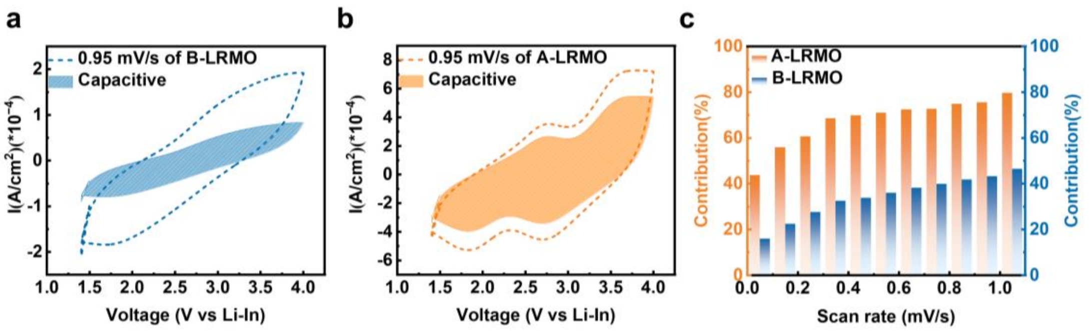

Figure S25. CV curves of capacitive current distinguished from total current at 0.95 mV s^{-1} for (a) B-LRMO and (b) A-LRMO samples accompanied with (c) corresponding capacitive contribution ratios comparison.

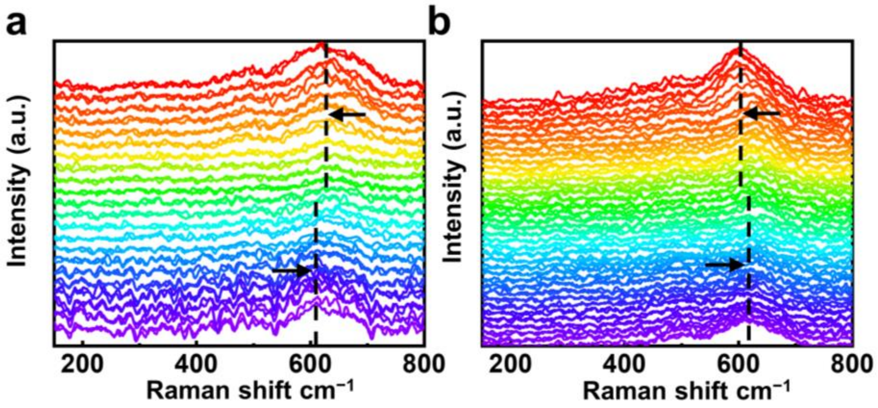

Figure S26. The signal variations of *operando* Raman characterizations for ASSLBs with (a) B-LRMO and A-LRMO cathodes.

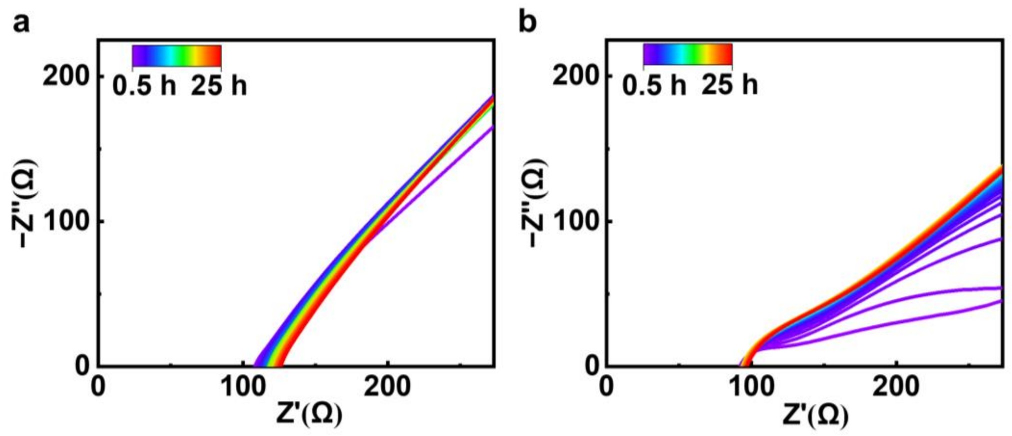

Figure S27. Magnification of Nyquist plots of (a) B-LRMO-LIC-VGCF/LPSC/Li-In and (b) A-LRMO-LIC-VGCF/LPSC/Li-In ASSLBs after charging up to and maintaining at 4.6 V vs. Li^{+}/Li under 0.1C at RT.

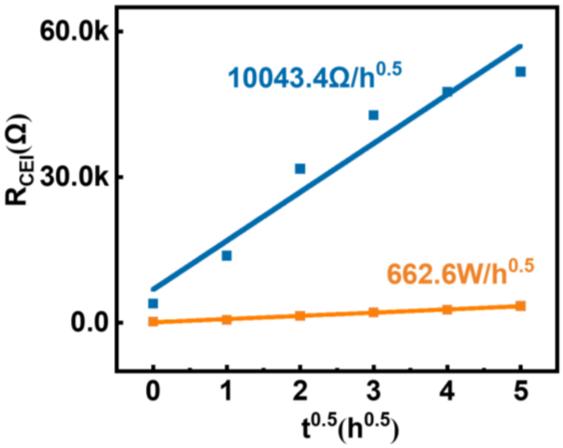

Figure S28. Comparison of interface resistance of RCEI change as a function of the square root of time ($t^{0.5}$) for highlighting differences between A-LRMO and B-LRMO cathodes.

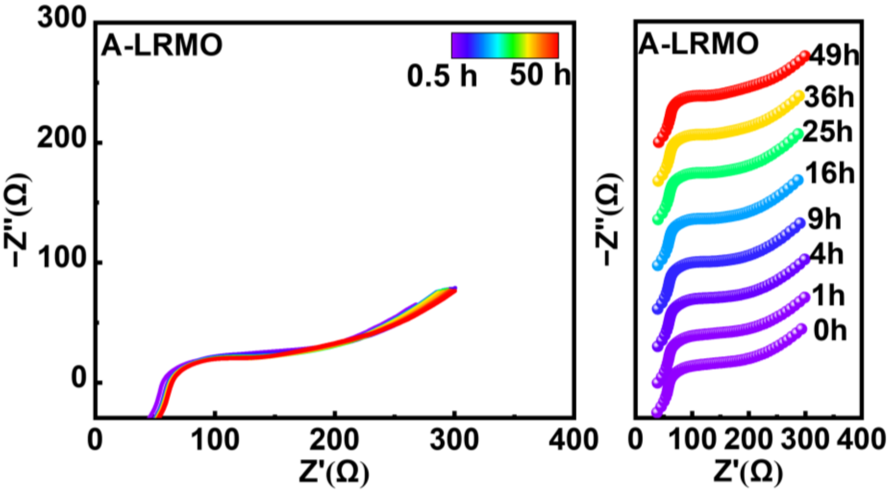

Figure S29. (a) Nyquist plots of A-LRMO-LIC-VGCF/LPSC/Li-In ASSLBs after charging up to 4.62 V vs. Li^+/Li under 0.1C at RT.

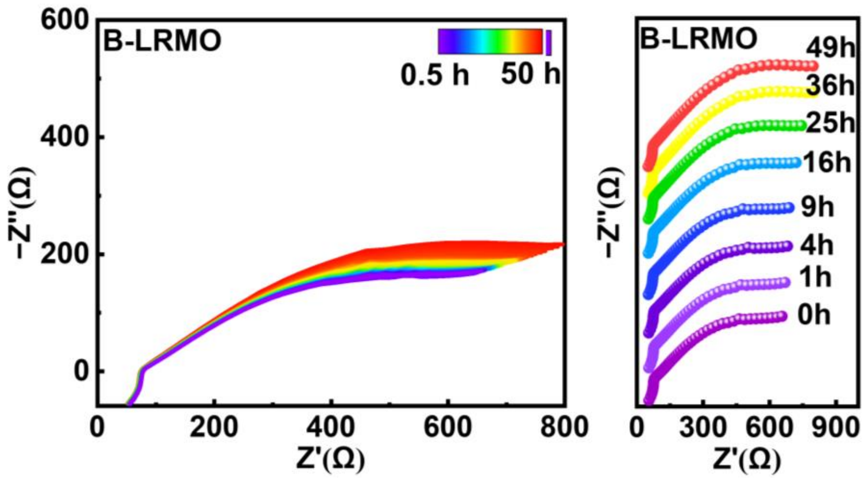

Figure S30. (a) Nyquist plots of B-LRMO-LIC-VGCF/LPSC/Li-In ASSLBs after charging up to 4.62 V vs. Li^{+}/Li under 0.1C at RT.

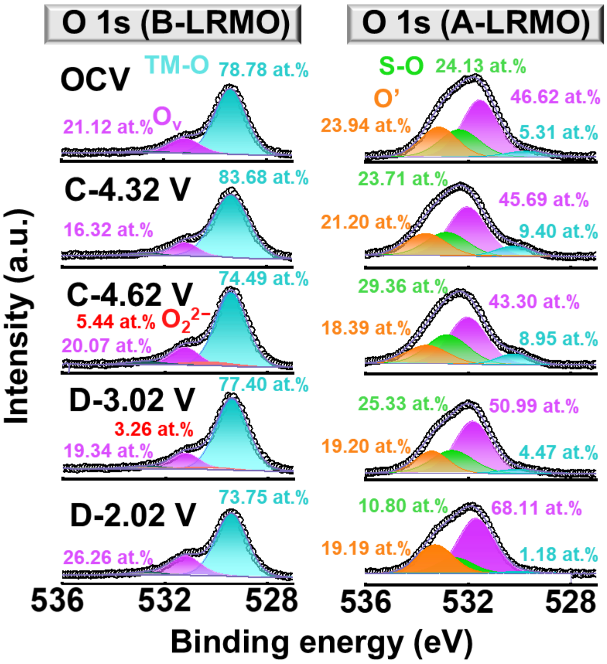

Figure S31. XPS spectra and curve fitting for O 1s of A-LRMO and B-LRMO cathodes within ASSLBs under different charge states within the range of 2.02-4.62 V vs. Li^+/Li.

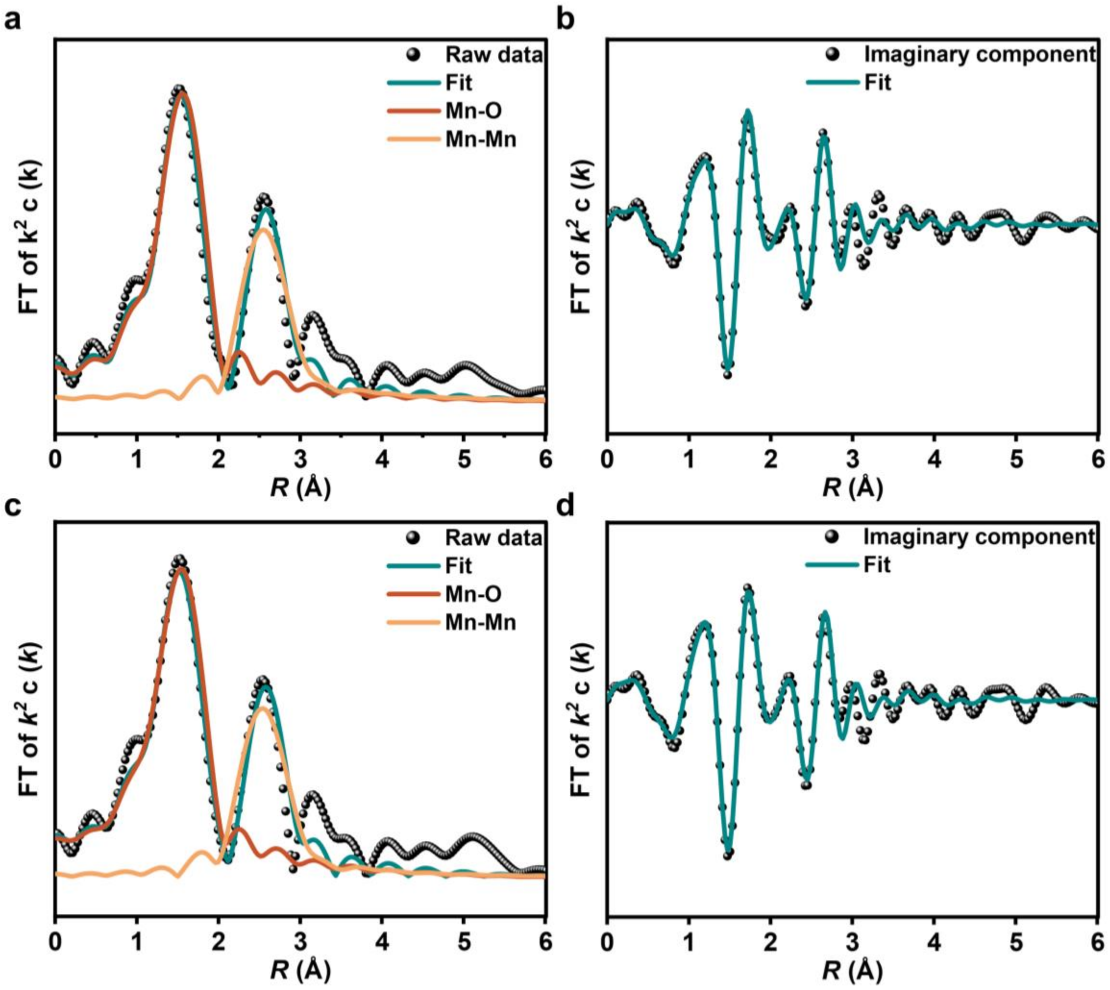

Figure S32. The EXAFS spectra of Mn K-edge experimental data and fitting r-results of B-LRMO cathodes within ASSLBs (a) before and (c) after cycling, accompanied with (b, d) corresponding imaginary components.

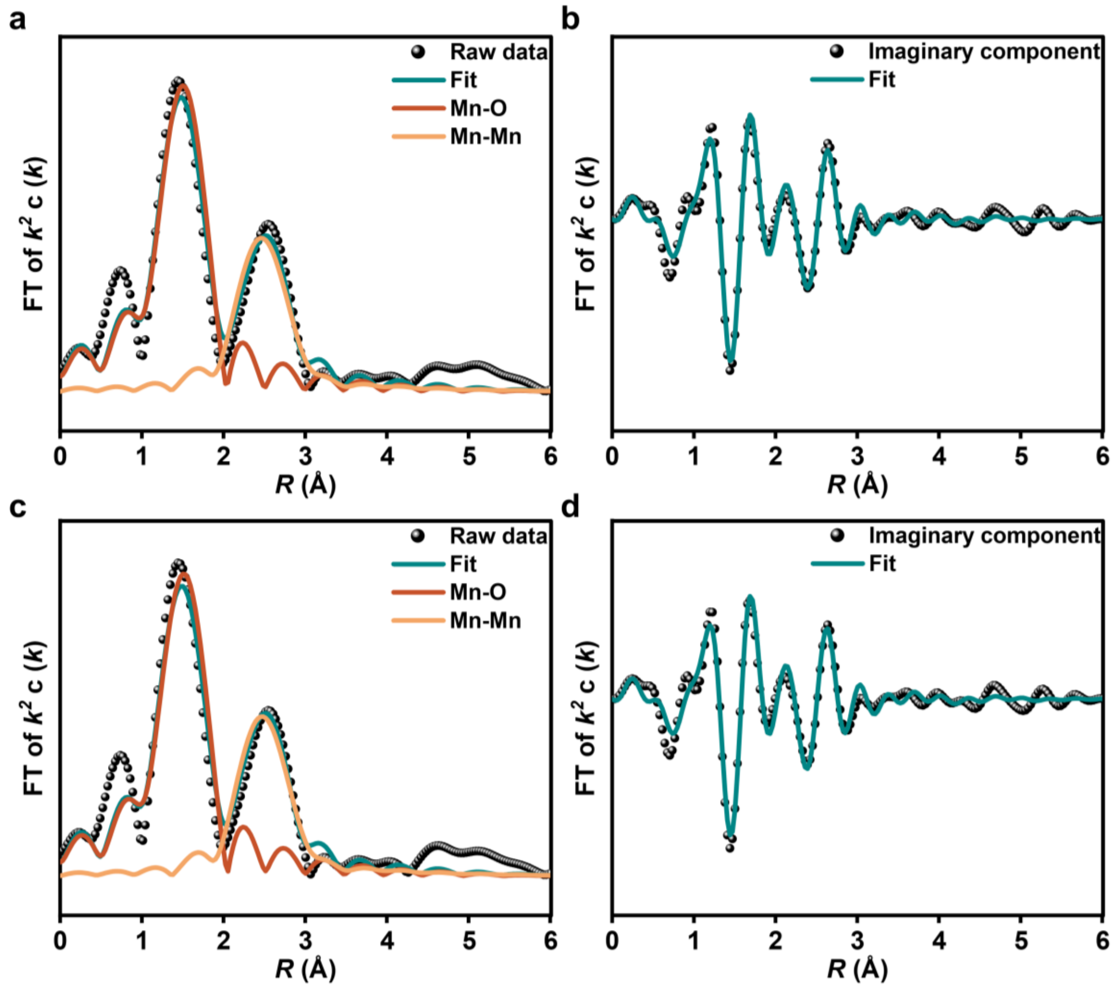

Figure S33. The EXAFS spectra of Mn K-edge experimental data and fitting r-results of A-LRMO cathodes within ASSLBs (a) before and (c) after cycling, accompanied with (b, d) corresponding imaginary components.

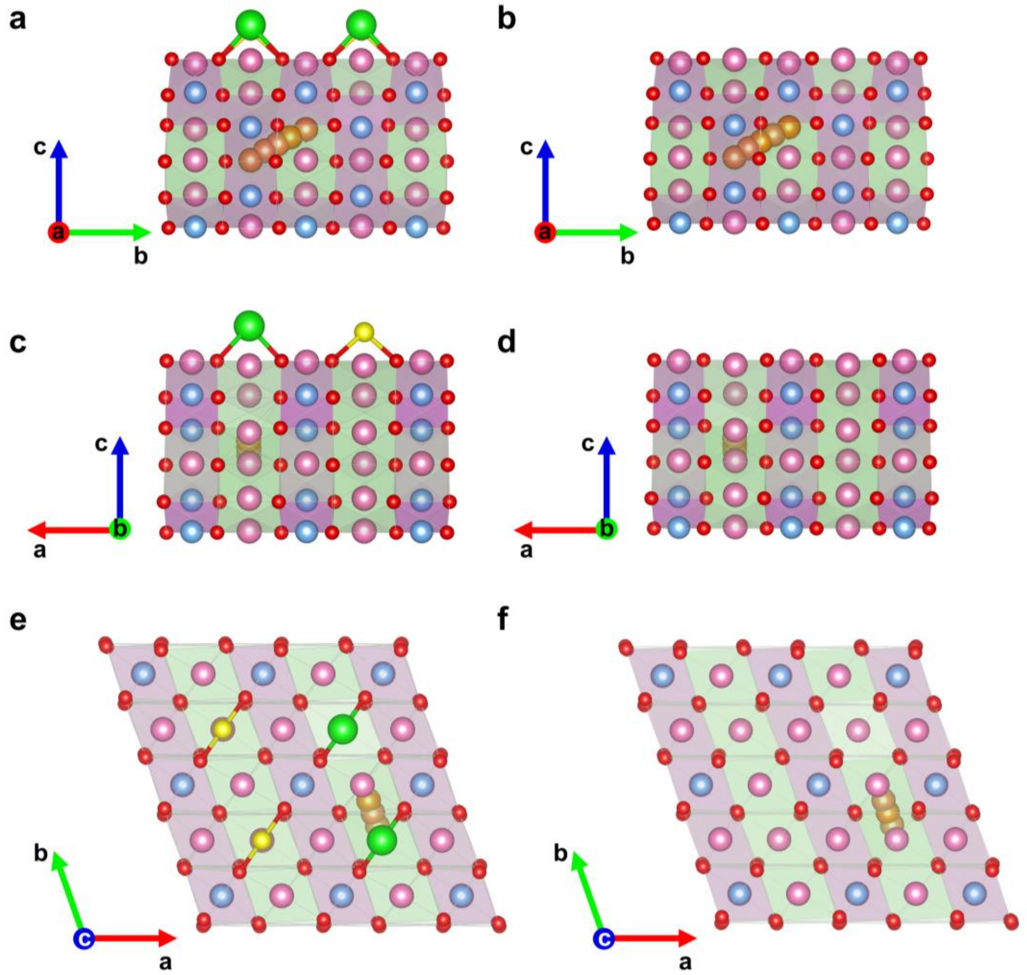

Figure S34. The observations of Zr, S co-doping LRMO and pristine LRMO lattice structures along (a, b) a, (c, d) b and (e, f) c axis within the path I mode.

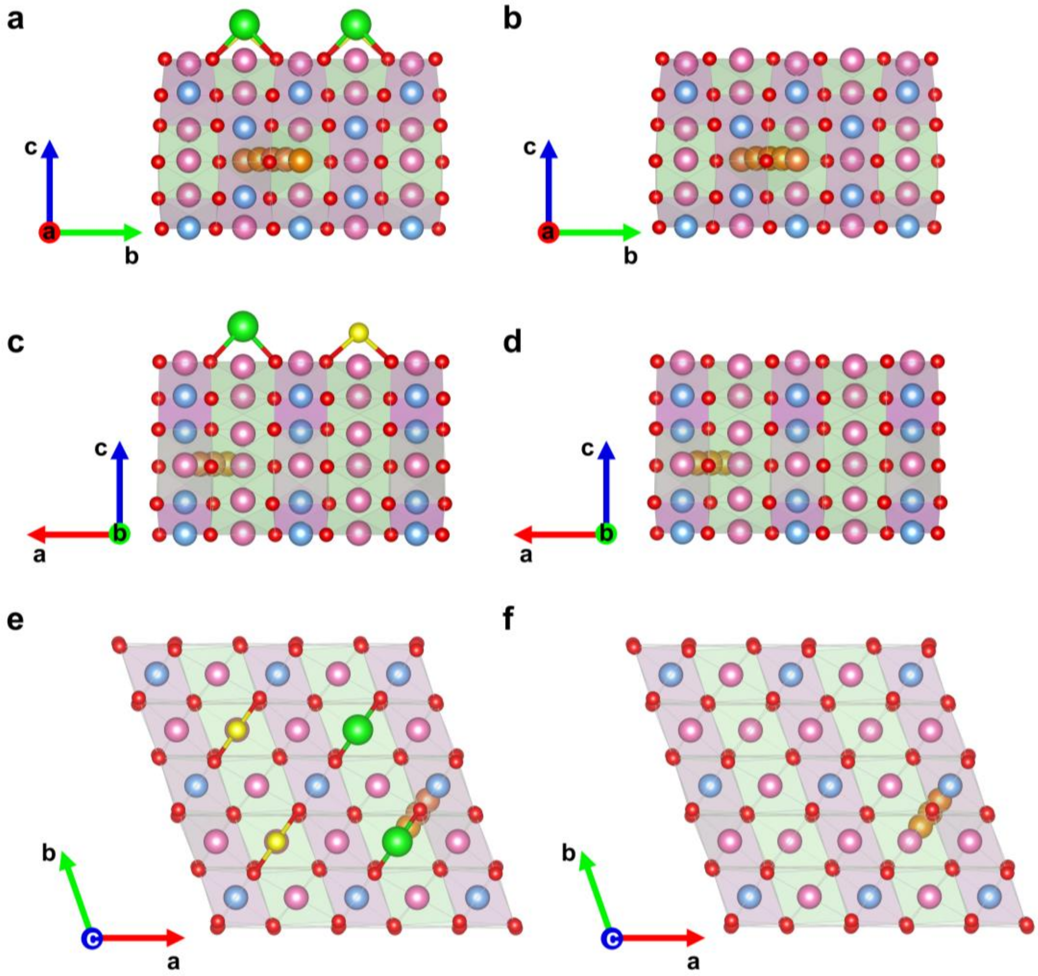

Figure S35. The observations of Zr, S co-doping LRMO and pristine LRMO lattice structures along (a, b) a, (c, d) b and (e, f) c axis within the path II mode.

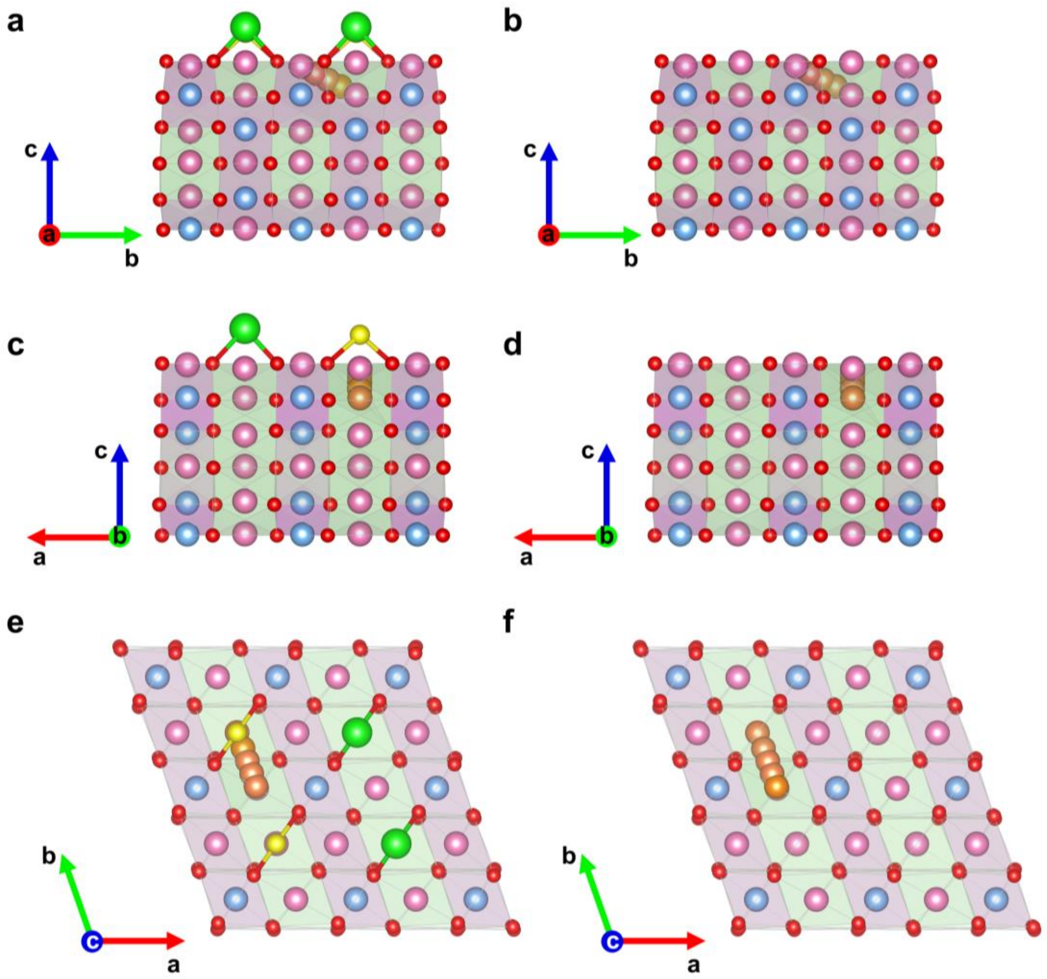

Figure S36. The observations of Zr, S co-doping LRMO and pristine LRMO lattice structures along (a, b) a, (c, d) b and (e, f) c axis within the path III mode.

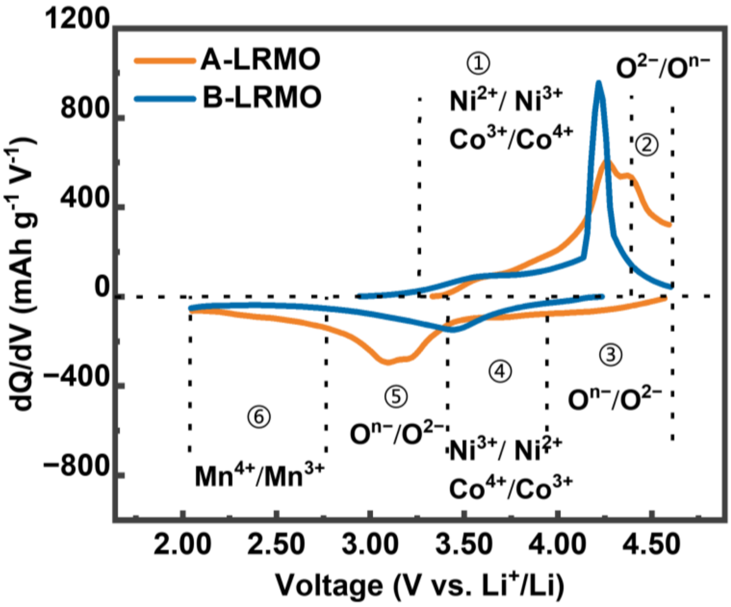

Figure S37. Initial dQ/dV charge/discharge curves of A-LRMO and B-LRMO cathodes.

Description: the dQ/dV curves reveal redox peaks corresponding to the evolution of transition metal ions and anionic O^{2-} within the LRMO cathode across different voltage ranges during charge and discharge.

During the charging process (to high voltage >4.4 V, region ②): The modified A-LRMO sample exhibits greater participation of anionic O^{2-} in charge compensation. In contrast, for the B-LRMO sample, the preferential oxidation of surface anions generates highly reactive O^{n-} species, which readily oxidize the surrounding LIC electrolyte. This leads to interface degradation and a sharp increase in resistance—consistent with GEIS and GITT data—thereby severely limiting the involvement of bulk anionic O^{2-} in charge compensation within the B-LRMO cathode.

During the discharging process: The reduction peaks in voltage regions ③ and ⑤ correspond to the reduction reaction of anionic O^{n-}. It is observed that the A-LRMO cathode exhibits more distinct anionic O^{n-} reduction peaks compared to B-LRMO,

indicating higher reversibility of the anionic O^{n-} redox couple in A-LRMO. Furthermore, in region ④, the reduction potential of transition metal ions for B-LRMO is significantly lower than that for A-LRMO, demonstrating that A-LRMO possesses lower polarization and higher stability.

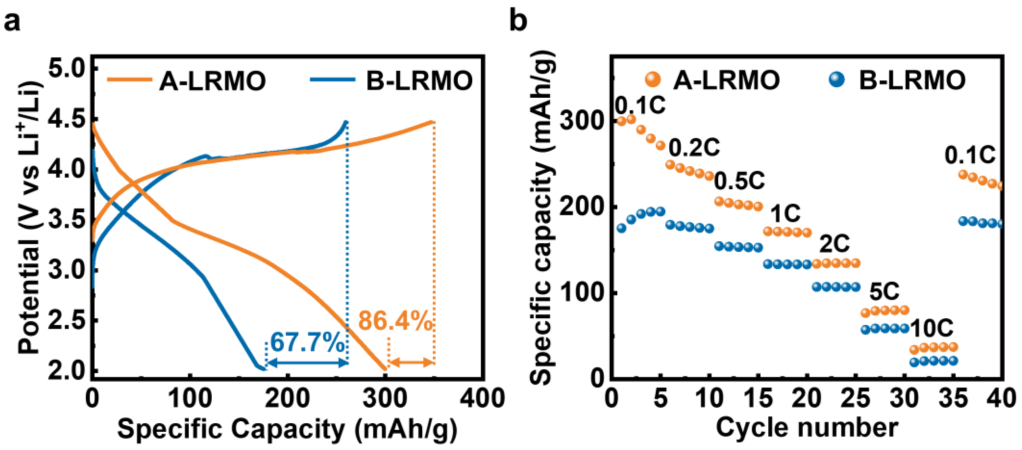

Figure S38. (a) The initial charge-discharge curves at 0.1C (1C = 350 mA g^{-1}) and (b) rate performances of A-LRMO-LIC VGCF/LPSC/Li-In and B-LRMO-LIC-VGCF/LPSC/Li-In ASSLBs at 60 °C.

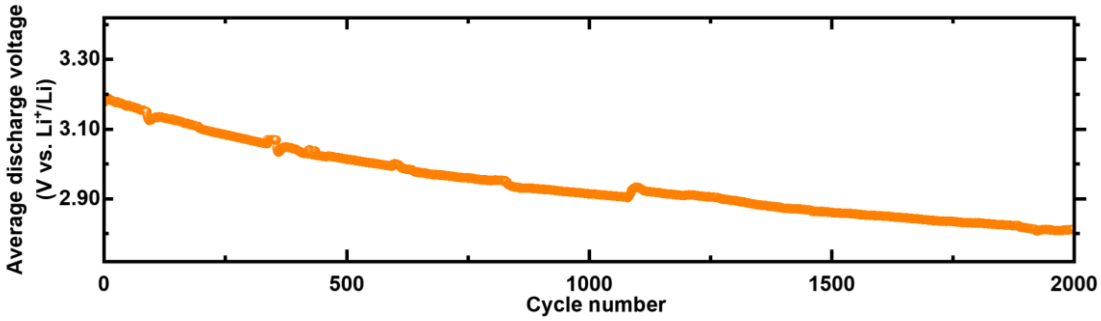

Figure S39. Average discharge voltage of long-term cycling at 1C for A-LRMO-LIC VGCF/LPSC/Li-In ASSLB based on mould cell at RT.

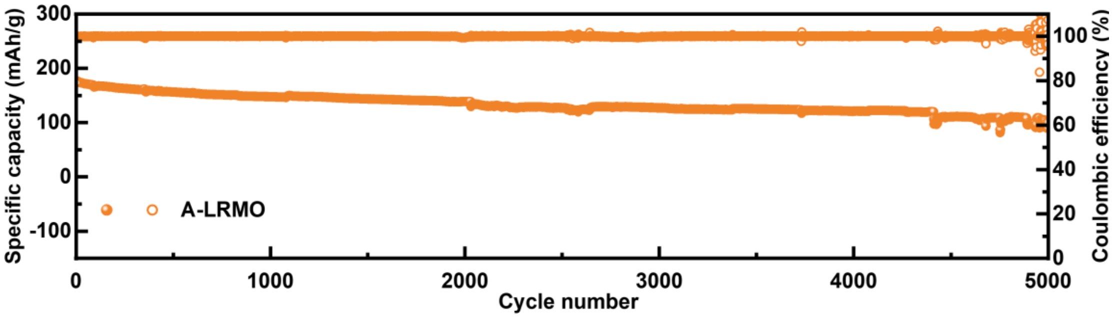

Figure S40. Long-term cycling stabilities at 1C of A-LRMO-LIC VGCF/LPSC/ ASSLB at RT.

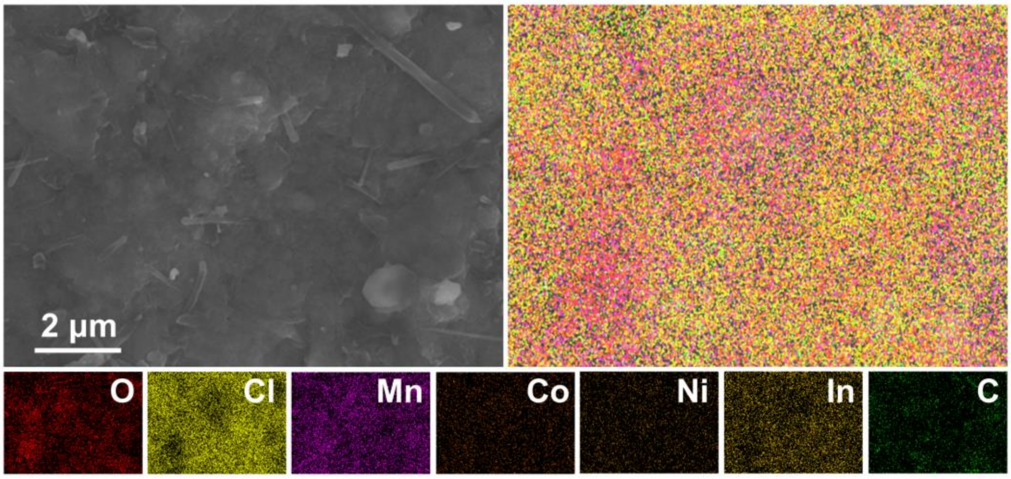

Figure S41. The SEM observations and EDS analyses of composite cathode within B-LRMO-LIC-VGCF/LPSC/Li-In ASSLB before cycling.

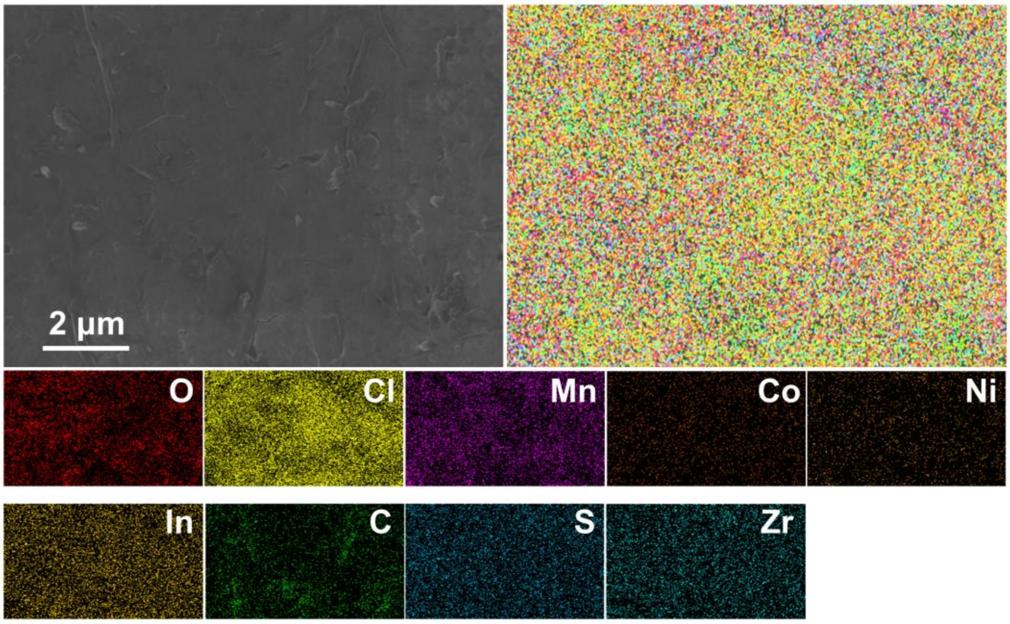

Figure S42. The SEM observations and EDS analyses of composite cathode within A-LRMO-LIC-VGCF/LPSC/Li-In ASSLB before cycling.

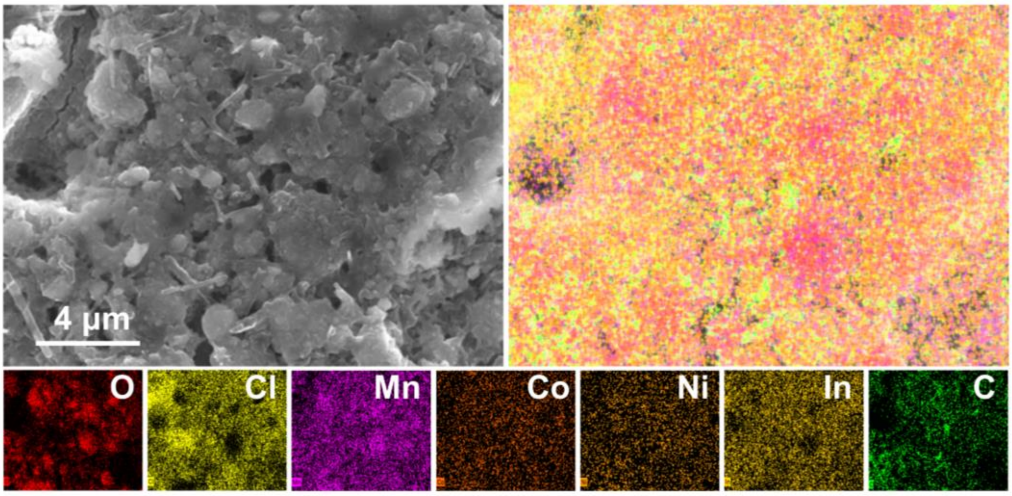

Figure S43. The SEM observations and EDS analyses of composite cathode within B-LRMO-LIC-VGCF/LPSC/Li-In ASSLB after cycling.

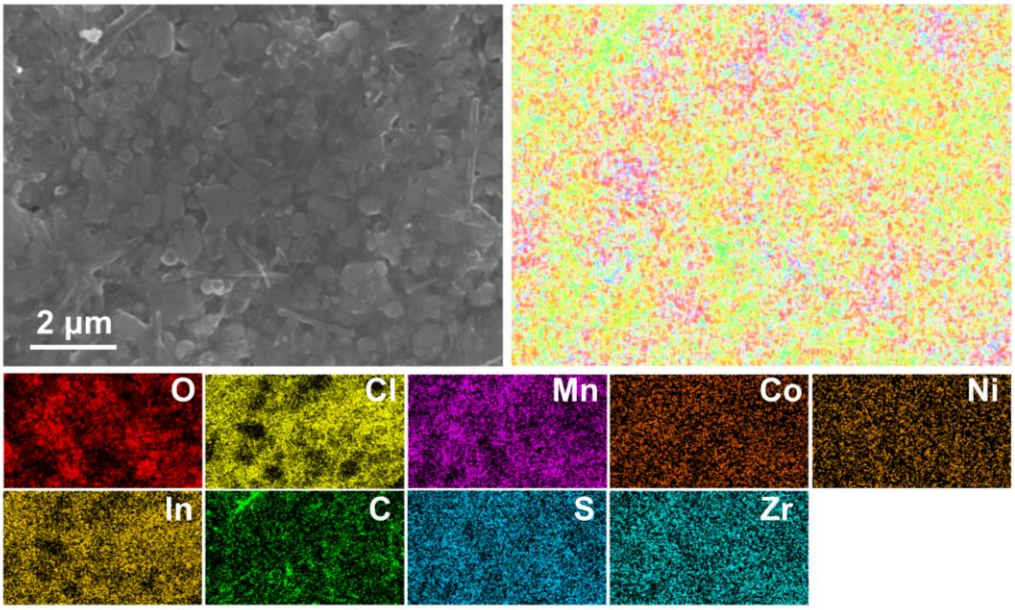

Figure S44. The SEM observations and EDS analyses of composite cathode within A-LRMO-LIC-VGCF/LPSC/Li-In ASSLB after cycling.

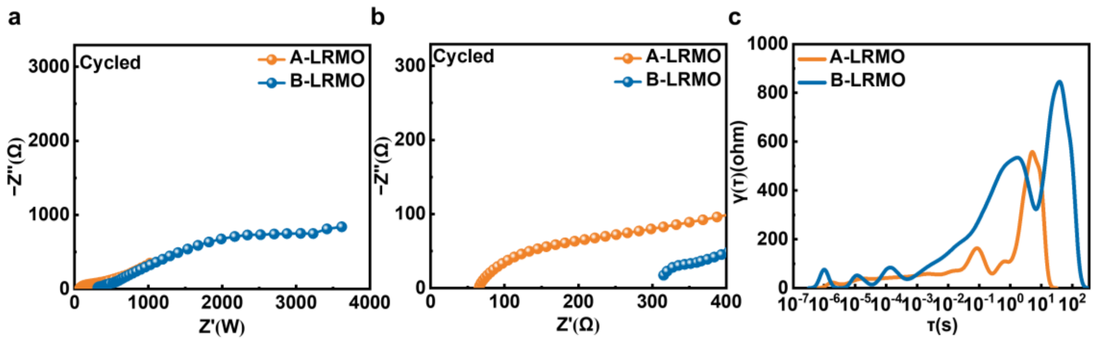

Figure S45. (a, b) Nyquist plots of A-LRMO-LIC-VGCF/LPSC/Li-In and B-LRMO-LIC-VGCF/LPSC/Li-In ASSLBs after cycling, accompanied with (c) corresponding DRT analyses.

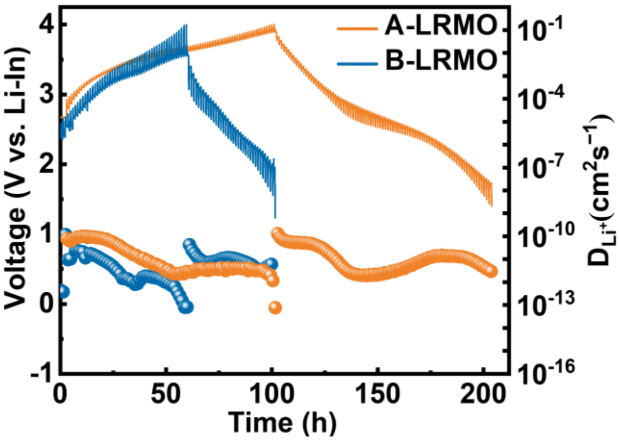

Figure S46. GITT potential curves of A-LRMO-LIC-VGCF/LPSC/Li-In and B-LRMO-LIC-VGCF/LPSC/Li-In ASSLBs after long-term cycling test.

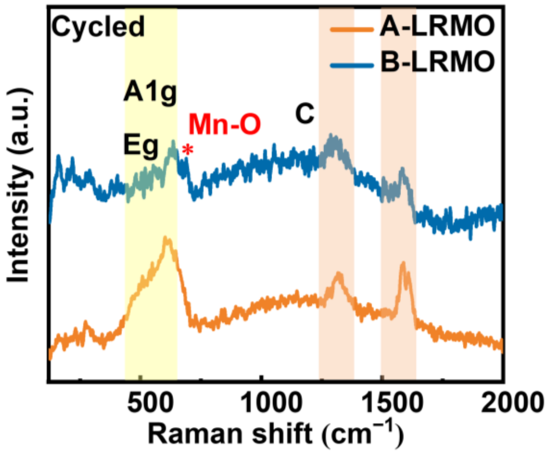

Figure S47. Raman spectra of composite cathodes within A-LRMO-LIC-VGCF/LPSC/Li-In and B-LRMO-LIC-VGCF/LPSC/Li-In ASSLBs after long-term cycling test.

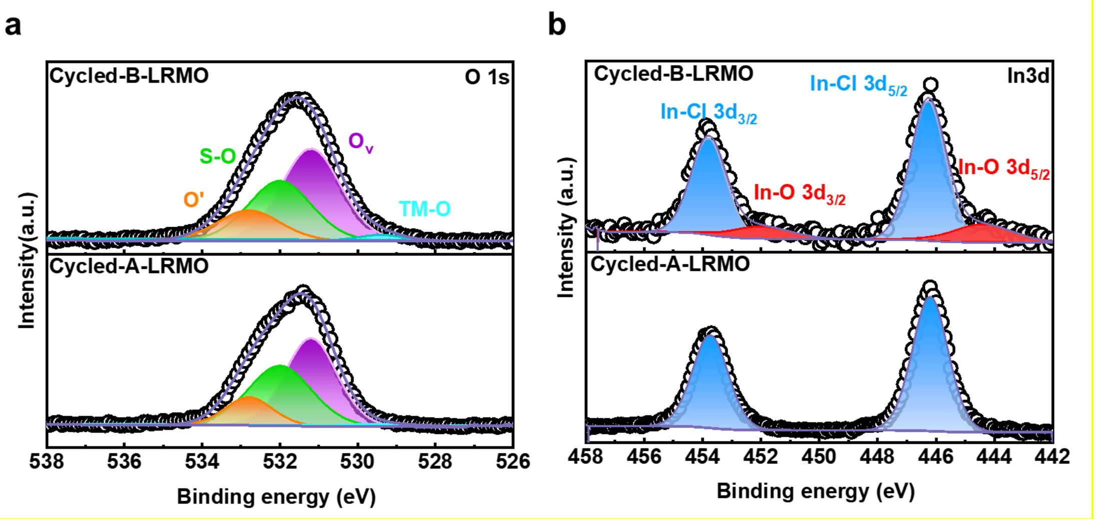

Figure S48. XPS spectra and curve fitting for (a) O 1s and (b) In 3d of composite cathodes within A-LRMO-LIC-VGCF/LPSC/Li-In and B-LRMO-LIC-VGCF/LPSC/Li-In ASSLBs after long-term cycling test.

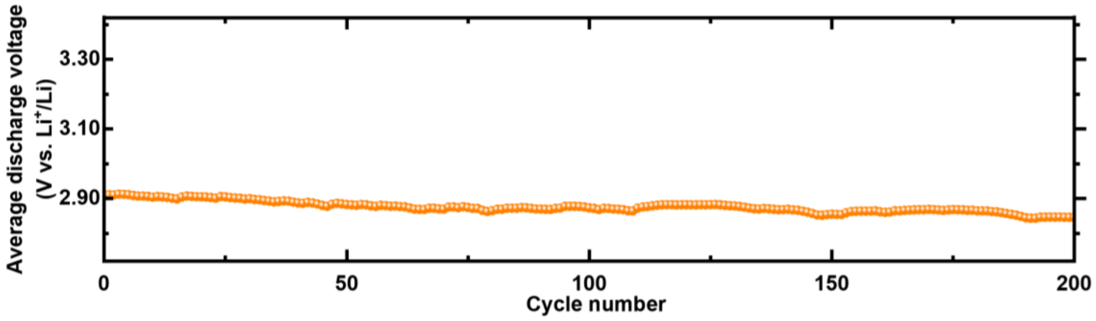

Figure S49. Average discharge voltage of long-term cycling at 0.2C for A-LRMO-LIC VGCF/LPSC/Li-In ASSLB based on pouch cell at RT.

Table S1. XPS binding energies with attributed species.

Spectra details	Binding energy (eV)	Attributed species
Zr	$3d_{3/2}$: 186.33<br>$3d_{5/2}$: 183.95	Zr-O ($Zr(SO_4)_2$)
	$3d_{3/2}$: 183.93<br>$3d_{5/2}$: 181.50	Zr-O (A-LRMO)
S	$2p_{1/2}$: 169.43<br>$2p_{3/2}$: 168.23	S-O (A-LRMO)
	$2p_{1/2}$: 169.57<br>$2p_{3/2}$: 168.27	S-O (SO_4^{2-})
	$2p_{1/2}$: 167.73<br>$2p_{3/2}$: 166.43	S-O (SO_3^{2-})
O	1s: 531.97	S-O (SO_4^{2-})
	1s: 532.85	absorbed O
	1s: 531.15	O vacancy
	1s: 529.47	lattice O (A-LRMO)
	1s: 529.25	lattice O (B-LRMO)
Mn	3s: 88.81, 84.10	A-LRMO
	3s: 88.71, 83.85	B-LRMO
In	$3d_{3/2}$: 453.8<br>$3d_{5/2}$: 446.2	In-Cl (LIC)
	$3d_{3/2}$: 452.2<br>$3d_{5/2}$: 444.8	In-O (In_2O_3)

Table S2. Voltage, current, resistance, thickness, area, and electronic conductivity value for current-time curves of the cathode-SE-VGCF composite (60:40:5 wt %) for A-LRMO and B-LRMO samples in a steel/composite/steel ion blocking cell.

Cathode/SE/VGCF	$V_{composite}$ (V)	$I_{composite}$ (mA)	$R_{composite}$ (Ω)	$L_{composite}$ (cm)	$A_{composite}$ (cm^2)	$\sigma_{composite}$ (S/cm)
A-LRMO	0.5V	0.00846	59.1016548	0.044	0.785	9.484E-04
B-LRMO	0.5V	0.00631	79.2393027	0.041	0.785	6.59E-04

Table S3. Voltage, current, resistance, thickness, area, and electronic conductivity value for current-time curves of the cathode-SE-VGCF composite (60:40:5 wt %) for A-LRMO, B-LRMO and LIC samples in a Li-In/LIC/composite/LIC/Li-In electron blocking cell.

Cathode/SE/VGCF	$V_{composite}$ (V)	$I_{composite}$ (mA)	$R_{composite}$ (Ω)	$L_{composite}$ (cm)	$A_{composite}$ (cm^2)	$\sigma_{composite}$ (S/cm)
A-LRMO	0.2V	0.00103912	192.4706	0.044	0.785	2.91E-04
B-LRMO	0.2V	0.000175385	265.3048	0.041	0.785	1.98E-04
LIC	0.2V	0.00228027	87.70891	0.140	0.785	2.03E-03

Table S4. Symbols and denoted meanings for CA|SE interface resistance in Equation 4.

Symbol	Meaning
S	Contact area
$\overline{\sigma_{CEI}}$	Average ionic conductivity of CA|SE interface layer
V_m	Average molar volume of CA|SE interface layer
x	Number of moles of Li extracted from LIC
F	Faraday's constant
$\overline{\sigma_{Li+}}$	Partial ionic conductivity of CA|SE interface layer
$\overline{\sigma_{e-}}$	Partial electronic conductivity of CA|SE interface layer
μ_{Li}	Difference in the chemical potential of lithium across the interface layer
t	Resting time
K, k'	Rate constant

Table S5. XPS binding energies for Zr 3d during charge/discharge process in Figure 3e.

**SOC**	**Zr^{4+} $3d_{3/2}$ (eV)**	**Zr^{4+} $3d_{5/2}$ (eV)**
OCV	183.8	181.5
C-4.32 V	184.6	182.3
C-4.62 V	184.5	182.2
D-3.02 V	184.4	182.1
D-2.02 V	184.0	181.7

Table S6. EXAFS curve-fitting results of B-LRMO and A-LRMO cathodes before and after cycling.

Sample	path	C.N.[a]	*R*(Å)[b]	σ^2(Å^2)[c]	△E_0(eV)[d]	*R* factor[e]
B-LRMO-pristine	Mn-O	6*	2.06±0.01	0.0012±0.0003	−5.16±1.68	0.0093
	Mn-Mn	2*	3.01±0.01	0.0068±0.0038		
B-LRMO-cycled	Mn-O	6*	2.05±0.01	0.0030±0.0006	−6.74±1.63	0.0095
	Mn-Mn	2*	2.88±0.02	0.0081±0.0038		
A-LRMO-pristine	Mn-O	6*	2.02±0.01	0.0062±0.0017	−7.91±1.57	0.0128
	Mn-Mn	2*	2.97±0.02	0.0073±0.0025		
A-LRMO-cycled	Mn-O	6*	2.01±0.01	0.0070±0.0021	−7.62±1.64	0.0141
	Mn-Mn	2*	2.97±0.01	0.0084±0.0030		

[a]C. N.: coordination number; [b]*R*: bond distance; [c]σ^2: Debye-Waller factors; [d]△E_0: the inner potential correction. [e]*R* factor: goodness of fit. The uncertainties of the parameters are also provided. *This parameter is fixed during EXAFS fitting.

***k* range.**

k range	11Mn	12Mn	13Mn	14Mn
	3.0-9.9	3.0-9.9	3.0-9.5	3.0-9.5

Table S7. Performances of ASSLBs described in previously reported representative papers compared with our work.

Methods	Precursor	Initial capacity (rate)/mA h g^{-1}	Initial CE (%)	Lifespan	C-Rate	average fading (%)
	MgO[6]	196.5 (0.2C)	78	200	0.2C	0.07
	Al_2O_3[7]	266.1 (0.1C)	88.7	100	0.1C	0.113
	TiO_2 [8]	254.6 (0.2C)	82.4	100	1C	0.204
	ZrO [9]$_2$	308.5 (0.1C)	95.38	170	0.2C	0.184
	CeO_2[10]	196.8 (1C)	80.2	200	1C	0.057
	Ta_2O_5[11]	292.9 (0.1C)	80.16	100	2C	0.147
	In_2O_3[12]	268.2 (0.1C)	72.5	200	1C	0.1
	$VOPO_4$[13]	260.5 (0.1C)	99.6	100	1C	0.078
Coating	$Li_{1.4}Al_{0.4}Ti_{1.6}(PO_4)_3$[14]	234.5 (0.1C)	-	100	0.2C	0.14
	$LiTi_2(PO_4)_3$[15]	262.1 (0.1C)	78.4	400	1C	0.03375
	$AlPO_4$-Li_3PO_4[16]	260.73 (0.1C)	84.96	200	0.2C	0.126
	AlF_3[17]	283.3 (0.1C)	88.3	200	1C	0.078
	TbF_3[18]	220.7 (0.1C)	80.43	300	1C	0.0163
	LaF_3[19]	256.1 (0.08 C)	-	100	0.5C	0.133
	PI/MWCNT[20]	238.7 (0.1C)	81.1	200	1C	0.135
	carbon[21]	281.7 (0.1C)	85.1	200	5C	0.1385
	Graphene quantum dot[22]	252.6 (0.2 C)	65.7	150	1C	-

	Crosslinked Hetero-Chain Polymeric[23]	268.6 (0.5C)	-	825	0.5C	0.0286
	Polyacrylonitrile[24]	291.1 (0.1 C)	83.1	275	0.5C	-
	Li_3PO_4[25]	199.7 (0.1C)	70.7	50	0.1C	0.524
	LiF[26]	208.4 (0.1C)	87.3	150	2C	0.123
	Li_2TiO_3[27]	276.5 (0.1C)	86.3	125	0.2C	0.0024
	Li_2ZrO_3[28]	275.7 (0.1 C)	-	200	1C	0.1541
	$LiNbO_3$[29]	280.5 (0.1 C)	63.6	100	0.5C	0.0853
	$LiAlO_2$[30]	245 (0.1 C)	82.28	300	1C	0.1099
	Mg^{2+}[31]	208.1 (0.2C)	67.46	200	0.2C	0.0886
	Na^{+}[32]	280.0 (0.1C)	91.2	200	1C	0.00025
	Ti^{4+}[33]	244.2 (0.05C)	-	100	0.2C	0.1272
	Zr^{4+}[34]	283.8 (0.2C)	77.38	100	0.4C	0.06
Doping	F^{-}[35]	275.3 (0.2C)	71.2	100	1C	0.181
	Se^{2-}[36]	293.5 (0.1C)	94.3	400	1C	0.029
	Mg^{2+}+V^{3+}[37]	209.2(0.1C)	75.34	200	0.5C	0.2812
	Ta^{5+}+Mo^{6+}[38]	243.2 (1C)	-	240	1C	0.0833
	Fe^{3+}+Cl^{-}[39]	232.4 (0.2C)	73.1	500	1C	0.0272
This work		291.0 (0.1C)	92.1	2000	1C	0.0091

Table S8. Performance comparison between previous representative works

Active materials	Area capacity (mAh·cm^{-2})	Average discharge voltage (V)	Energy (Wh)	Anode Type	Full battery quality (g)	Energy density (Wh/kg)	Battery Type
A-LRMO	8.60	2.60	0.6130	Li-In	3.2008	191.51	Pouch cell
A-LRMO	8.60	3.22	0.6130	Li	1.4589	420.18	Pouch cell
A-LRMO	2.55	2.68	0.0066	Li-In	0.3120	21.15	Model cell
LBO-LRMO [40]	1.69785	2.65	0.0035	Li-In	0.2858	12.24	Model cell
5W&LRMO[41]	3.15	2.8	0.0069	Li-In	0.2086	33.08	Model cell
S-LRMO[42]	1.10	2.65	0.0023	Li-In	0.1848	12.44	Model cell
S-LRMO[42]	2.90	2.75	0.0063	Li-In	0.1964	32.07	Model cell

3. References in supporting information

[1] F. Hippauf, B. Schumm, S. Doerfler, H. Althues, S. Fujiki, T. Shiratsuchi, T. Tsujimura, Y. Aihara, S. Kaskel, Energy Storage Materials 2019, 21, 390.

[2] Y.-G. Lee, S. Fujiki, C. Jung, N. Suzuki, N. Yashiro, R. Omoda, D.-S. Ko, T. Shiratsuchi, T. Sugimoto, S. Ryu, J. H. Ku, T. Watanabe, Y. Park, Y. Aihara, D. Im, I. T. Han, Nature Energy 2020, 5, 299.

[3] B. Cao, H. Liu, P. Zhang, N. Sun, B. Zheng, Y. Li, H. Du, B. Xu, Advanced Functional Materials 2021, 31, 2102126.

[4] P. E. Blöchl, Physical review B 1994, 50, 17953; J. P. Perdew, K. Burke, M. Ernzerhof, Physical review letters 1996, 77, 3865.

[5] G. Henkelman, B. P. Uberuaga, H. Jónsson, The Journal of chemical physics 2000, 113, 9901.

[6] X. Cui, J. Zhang, C. Li, X. Cai, J. Zhou, H. Ding, J. Bai, N. Zhang, M. Wu, J. Yan, Y. Zhang, S. Li, Acta Materialia 2025, 290, 120951.

[7] X. Sun, Y. Du, W. Zhang, M. Jin, J. Li, P. Zhang, J Alloy Compd 2025, 1031, 180990.

[8] X. Ran, J. Tao, Z. Chen, Z. Yan, Y. Yang, J. Li, Y. Lin, Z. Huang, Electrochimica Acta 2020, 353, 135959.

[9] S. Dong, X. He, Q. Xu, L. Ma, C. Hai, Y. Zhou, Langmuir 2023, 39, 7723.

[10] X.-Y. Feng, M.-J. Ran, M.-M. Yuan, M.-T. Wei, L. Wu, Z.-Y. Hu, L.-H. Chen, Y. Li, B.-L. Su, ACS Applied Nano Materials 2024, 7, 13173.

[11] X. Ding, Q. Liu, H. Zhu, Journal of Solid State Electrochemistry 2022, 26, 1115.

[12] M. Yu, X. Wei, X. Min, A. Yuan, J. Xu, Mater Chem Phys 2022, 286, 126228.

[13] X. Xie, H. Li, S. Cao, C. Wu, Z. Li, B. Chang, G. Chen, X. Guo, T. Wu, X. Wang, Energ Fuel 2021, 35, 14148.

[14] X.-w. Lai, G.-r. Hu, Z.-d. Peng, Y.-b. Cao, K. Du, Y.-x. Liu, Journal of Central South University 2022, 29, 1463.

[15] L. Sai, Z. Dai, Z. Wang, H. Zhao, Y. Bai, Journal of Power Sources 2024, 613, 234870.

[16] Y. Wang, W. Yu, L. Zhao, A. Wu, A. Li, X. Dong, H. Huang, Electrochimica Acta 2023, 462, 142664.

[17] J. Zhang, D. Zhang, Z. Wang, F. Zheng, R. Zhong, R. Hong, J Mater Sci 2023, 58, 4525.

[18] Z. Li, W. Song, D. Zhang, Q. Wang, H. Sun, Q. Sun, B. Wang, Acs Appl Mater Inter 2024, 16, 25210.

[19] J. Liu, F. Bei, L. Wen, Z. Zheng, B. Zhang, Q. Han, L. Wang, Y. Wu, X. He, Electrochimica Acta 2024, 479, 143882.

[20] Y. Wang, P. Fan, B. Liu, X. Li, Y. Fu, G. Du, J. Liu, S. Chen, H. Sun, ACS Omega 2025, 10, 27415.

[21] B. Ramasubramanian, R. Prasada Rao, G. K. Dalapati, S. Adams, S. Ramakrishna, ACS Applied Energy Materials 2024, 7, 3018.

[22] M. Yu, X. Wei, X. Min, A. Yuan, J. Xu, Energ Fuel 2022, 36, 5502.

[23] W.-h. Hou, Q. Feng, C. Liu, X. Zhang, J. Yue, Q. Tian, S. Wu, Y. Ou, P. Zhou, Y.

Xia, Y. Wang, X. Song, H. Zhou, Y. Lu, S. Yan, K. Liu, Adv Mater 2025, 37, 2503893.
[24]C. Zheng, Z. Yang, J. Feng, J. Zhong, Z. Wei, J. Li, J Mater Chem A 2022, 10, 16046.
[25]Y. Sun, X. Zhang, J. Cheng, M. Guo, X. Li, C. Wang, L. Sun, J. Yan, Ionics 2023, 29, 2141.
[26]C. Fu, Y. Zhou, G. Shen, H. Wang, Y. Wang, J. Shen, J. Fan, Z. Sun, Energy Materials and Devices 2025, 3, 9370065.
[27]J. Liu, Z. Wu, M. Yu, H. Hu, Y. Zhang, K. Zhang, Z. Du, F. Cheng, J. Chen, Small 2022, 18, 2106337.
[28]Z. He, J. Li, Z. Luo, Z. Zhou, X. Jiang, J. Zheng, Y. Li, J. Mao, K. Dai, C. Yan, Z. Sun, Acs Appl Mater Inter 2021, 13, 49390.
[29]Z. Li, M. Yuan, H. Liu, J. Liu, S. Xie, T. Wang, J. Yan, Journal of Materials Research 2022, 37, 3831.
[30]W. He, C. Zhang, M. Wang, B. Wei, Y. Zhu, J. Wu, C. Liang, L. Chen, P. Wang, W. Wei, Adv Funct Mater 2022, 32, 2200322.
[31]X. Cui, J. Zhou, H. Ding, X. Cai, J. Zhang, X. Hu, J. Zhang, X. Li, J. Zhu, N. Zhang, S. Li, Journal of Power Sources 2024, 623, 235458.
[32]S. Cao, J. Chen, H. Li, Z. Li, C. Guo, G. Chen, X. Guo, X. Wang, Journal of Power Sources 2023, 555, 232398.
[33]K. Zhang, Y. Tian, X. Chen, S. Hu, Z. Jian, Chin. Chem. Lett. 2024, 35, 108308.
[34]G. Wang, C. Xie, H. Wang, Q. Li, F. Xia, W. Zeng, H. Peng, G. Van Tendeloo, G. Tan, J. Tian, J. Wu, Adv Funct Mater 2024, 34, 2313672.
[35]L. Di, C. Yufang, S. Weiwei, X. Wei, Y. Shuaiyu, L. Shiqiang, Z. Lanlan, Z. Yanshuang, Y. Tianyan, X. Peitao, Z. Chunman, Adv Energy Mater 2023, 13, 2301765.
[36]J. Chen, H. Chen, W. Deng, X. Gao, S. Yin, Y. Mei, S. Zhang, L. Ni, J. Gao, H. Liu, Y. Tian, L. Yang, X. Deng, G. Zou, H. Hou, J. Xie, X. Ji, Energy Storage Materials 2022, 51, 671.
[37]J. Zhou, N. Zhang, X. Cai, J. Zhang, H. Dou, J. Zhang, J. Zhu, Y. Zhao, Y. Wang, M. Wang, D. Zhao, S. Li, X. Cui, Materials Today Energy 2024, 46, 101728.
[38]J. Yang, Y. Chen, Y. Li, X. Xi, J. Zheng, Y. Zhu, Y. Xiong, S. Liu, Acs Appl Mater Inter 2021, 13, 25981.
[39]L. Nie, Z. Wang, X. Zhao, S. Chen, Y. He, H. Zhao, T. Gao, Y. Zhang, L. Dong, F. Kim, Y. Yu, W. Liu, Nano Lett 2021, 21, 8370.
[40]S. Sun, C.-Z. Zhao, G.-Y. Liu, S.-C. Wang, Z.-H. Fu, W.-J. Kong, J.-L. Li, X. Chen, X. Zhao, Q. Zhang, Adv Mater 2025, 37, 2414195.
[41]W.-J. Kong, C.-Z. Zhao, L. Shen, S. Sun, X.-Y. Huang, P. Xu, Y. Lu, W.-Z. Huang, J.-L. Li, J.-Q. Huang, Q. Zhang, J Am Chem Soc 2024, 146, 28190.
[42]S. Sun, C.-Z. Zhao, H. Yuan, Z.-H. Fu, X. Chen, Y. Lu, Y.-F. Li, J.-K. Hu, J. Dong, J.-Q. Huang, M. Ouyang, Q. Zhang, Science Advances, 8, eadd5189.
